# A Conserved Role for *p48* Homologs in Protecting Dopaminergic Neurons from Oxidative Stress

**DOI:** 10.1371/journal.pgen.1004718

**Published:** 2014-10-23

**Authors:** Peter Bou Dib, Bettina Gnägi, Fiona Daly, Virginie Sabado, Damla Tas, Dominique A. Glauser, Peter Meister, Emi Nagoshi

**Affiliations:** 1 Institute of Cell Biology, University of Bern, Bern, Switzerland; 2 Graduate School for Cellular and Biomedical Sciences, University of Bern, Bern, Switzerland; 3 Department of Genetics and Evolution, University of Geneva, Sciences III, Geneva, Switzerland; 4 Department of Biology/Zoology, University of Fribourg, Chemin du Musée, Fribourg, Switzerland; 5 PRESTO, Japan Science and Technology Agency, Saitama, Japan; Stanford University School of Medicine, United States of America

## Abstract

Parkinson's disease (PD) is the most common neurodegenerative movement disorder characterized by the progressive loss of dopaminergic (DA) neurons. Both environmental and genetic factors are thought to contribute to the pathogenesis of PD. Although several genes linked to rare familial PD have been identified, endogenous risk factors for sporadic PD, which account for the majority of PD cases, remain largely unknown. Genome-wide association studies have identified many single nucleotide polymorphisms associated with sporadic PD in neurodevelopmental genes including the transcription factor *p48*/*ptf1a*. Here we investigate whether *p48* plays a role in the survival of DA neurons in *Drosophila melanogaster* and *Caenorhabditis elegans*. We show that a *Drosophila p48* homolog, *48-related-2* (*Fer2*), is expressed in and required for the development and survival of DA neurons in the protocerebral anterior medial (PAM) cluster. Loss of *Fer2* expression in adulthood causes progressive PAM neuron degeneration in aging flies along with mitochondrial dysfunction and elevated reactive oxygen species (ROS) production, leading to the progressive locomotor deficits. The oxidative stress challenge upregulates *Fer2* expression and exacerbates the PAM neuron degeneration in *Fer2* loss-of-function mutants. *hlh-13*, the worm homolog of *p48*, is also expressed in DA neurons. Unlike the fly counterpart, *hlh-13* loss-of-function does not impair development or survival of DA neurons under normal growth conditions. Yet, similar to *Fer2*, *hlh-13* expression is upregulated upon an acute oxidative challenge and is required for the survival of DA neurons under oxidative stress in adult worms. Taken together, our results indicate that *p48* homologs share a role in protecting DA neurons from oxidative stress and degeneration, and suggest that loss-of-function of *p48* homologs in flies and worms provides novel tools to study gene-environmental interactions affecting DA neuron survival.

## Author Summary

Parkinson's disease is a common movement disorder with no known cure. Its characteristic motor symptoms are primarily caused by the progressive loss of midbrain dopaminergic neurons. Although studies have shown that various environmental and genetic factors both contribute to the development of the disease, the underlying mechanisms remain unknown. Here we use powerful invertebrate model organisms, fruit flies and nematode worms, and identify a new gene required for the survival of dopaminergic neurons. We show that homologs of the *p48/ptf1-a* gene in both flies and worms are expressed in dopaminergic neurons and mutations in *p48* increase the susceptibility of dopaminergic neuron death when animals are under oxidative stress. Importantly, genetic variations in *p48* in humans have been detected in the sporadic Parkinson's disease patients, indicating the possibility that similar mechanism might play a role in the death of dopaminergic neurons in humans. Oxidative stress has been regarded as a major pathogenic factor for Parkinson's disease. Our results add evidence to the link between oxidative stress and neurodegeneration, and suggest that *p48* mutant flies and worms can be used to study mechanisms of neurodegeneration in Parkinson's disease.

## Introduction

Dopaminergic (DA) neurons play critical roles in motor control, cognition and motivation and are affected in many neurological and psychiatric disorders [1], [2], [3], [4]. The progressive degeneration of DA neurons in the substantia nigra pars compacta (SNc) is a principal pathological feature of Parkinson's disease (PD). PD is the most prevalent neurodegenerative movement disorder, for which no preventive or restorative therapies are available [5], [6]. The discovery of the genes associated with the rare familial forms of PD has led to the development of many animal models and advanced the understanding of PD pathogenesis. However, the majority of PD cases are sporadic and likely caused by a combination of environmental factors, such as pesticide exposure, and endogenous risk factors. These endogenous risk factors remain largely unknown. A recent meta-analysis on genome-wide association studies (GWAS) for PD showed that SNPs in the genes involved in multiple aspects of neural development are highly represented in sporadic PD patients [7], suggesting that genetic variations in these pathways may contribute to PD susceptibility. Indeed, several studies in mammals have shown the critical roles of developmental genes, such as *Engrailed1*, *foxa2* and *Nurr1*, in the survival of DA neurons in old age [8], [9], [10], [11]. The identification and characterization of such genes may yield a better molecular understanding of adult-onset neurodegeneration in PD.

The nervous system in invertebrate model organisms such as *Drosophila* and *C.elegans* shares many features with its mammalian counterpart and offers a powerful tool to study neural development and neurodegeneration. *Drosophila* DA neurons comprise multiple subclasses, some of which play roles similar to those played by the DA neurons in mammals, such as reward signaling and sleep regulation [12], [13]. The nematode *C.elegans* has 8 DA neurons, which are thought to have mechanosensory functions and have been shown to play a role in the modulation of locomotion [14]. Despite advances in anatomical and functional characterization, the mechanisms underlying the development and maintenance of DA neurons in flies and worms are poorly understood. *Drosophila Fer2*, a homolog of mammalian *p48/ptf1a*, belongs to the bHLH-transcription factor family, which is often involved in neurogenesis and neural subtype specification. The mammalian *p48* gene is a critical regulator for neural tube development [15], in which a candidate causal SNP for PD has been detected [7,16, The database of Genotypes and Phenotypes (dbGaP; NCBI)]. Previously, we showed that *Fer2* is required for the development of a subclass of circadian clock neurons, ventral Lateral Neurons (LNvs) [17]. Here we characterized additional roles of *Fer2* to better understand the genetic mechanisms of neuronal subtype development and maintenance. We unexpectedly found that *Fer2* is required for the development and maintenance of a subclass of DA neurons important for locomotion. *Fer2* exerts its neuroprotective role in adulthood in the oxidative stress response, and loss of *Fer2* expression in adulthood causes adult-onset progressive degeneration of these DA neurons. We further demonstrated that the *C. elegans* homolog of *p48*, *hlh-13*, is also required for the survival of DA neurons in adult worms under oxidative stress. Collectively, our results established a conserved role of *p48* homologs in protecting DA neurons from oxidative stress and degeneration.

## Results

### *Fer2* is required for the startle-induced climbing ability

**Figure 1:**
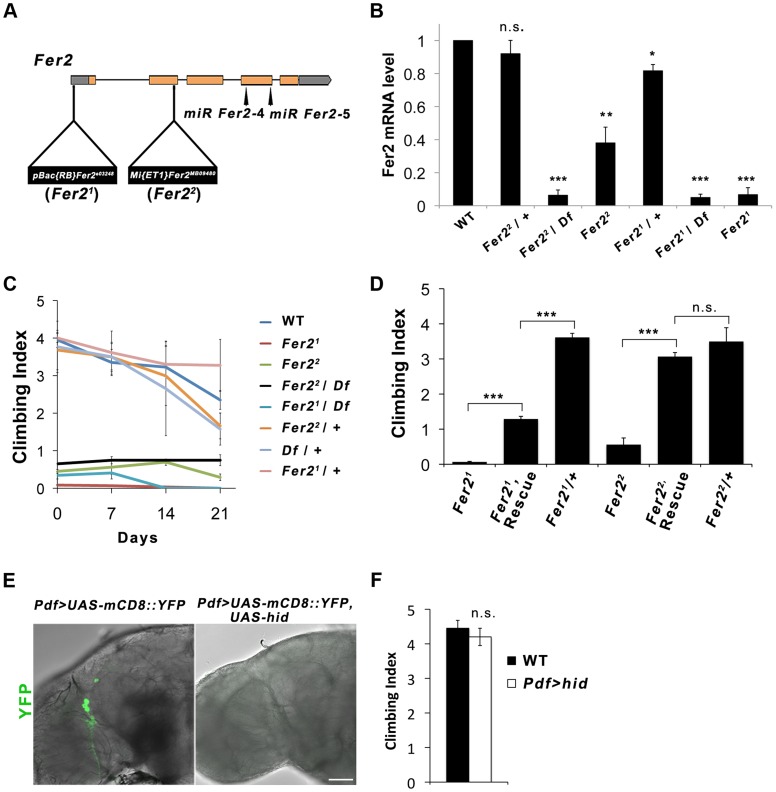
*Fer2* loss-of-function mutation impairs the startle-induced climbing ability. (A) Schematic of the *Fer2* mutant alleles and the *Fer2* miRNA target sites (*miR Fer2-4*, *miR Fer2-5*). Gray boxes are 5′ and 3′ UTRs, orange boxes are *Fer2* gene exons. (B) *Fer2* mRNA levels in the fly heads normalized to the level in *w^1118^* (WT). Mean ± SEM from 3 independent experiments. *p<0.05, ** p<0.01, *** p<0.001. n.s., no statistical significance. (C) Climbing abilities of *Fer2* mutants and control flies tested weekly. Mean Climbing Index from ± SEM. (D) *Fer2*> *Fer2-FLAG* (Rescue) significantly improved the impaired climbing ability of the *Fer2^1^* and *Fer2^2^* flies (7-day-old). Mean ± SEM. ***p<0.001. (E) PDF-positive LNvs visualized by expressing *UAS-mCD8::YFP* with *Pdf-GAL4* in the wild-type (*w^1118^*) and in the flies expressing *UAS-hid*. Error bar, 50 µm. (F) Climbing ability of the wild-type and LNv-ablated flies (*Pdf > hid*) at day 4. Mean ± SEM.

*Drosophila Fer2^e03248^* (henceforth referred to as *Fer2^1^*) mutation was induced by the insertion of *PBac*{*RB*} into the *Fer2* 5′UTR [18]. The *Fer2^MB09480^* (henceforth called *Fer2^2^*) allele has a *Mi*{*ET1*} transposon insertion in the 3′ end of the second exon [19] (Fig. 1A). To molecularly characterize the *Fer2* mutant alleles, we determined the *Fer2* mRNA levels using quantitative real-time PCR (qPCR). Consistent with our previous results, *Fer2* mRNA expression of the *Fer2^1^* homozygotes was approximately 5% of the wild-type level [17]. In the *Fer2^2^* homozygous flies, *Fer2* mRNA expression was reduced to about 40% of the wild-type level. Thus, *Fer2^1^* is an extreme hypomorphic allele, whereas *Fer2^2^* is a milder hypomorph. *Fer2* mRNA expression of both *Fer2^1^*/+ and *Fer2* ^2^/+ flies was only slightly reduced relative to the wild-type level, suggesting that the loss of one copy of a *Fer2* gene is compensated at the mRNA level by transcriptional or post-transcriptional mechanisms (Fig. 1B). Compensation of gene dose has been observed widely in both *Drosophila* and mammals [20], [21], [22]. Therefore, *Fer2* is a haplosufficient gene and *Fer2* heterozygous mutants are expected to be phenotypically wild-type.

We noticed that *Fer2* mutant flies tend to climb up the walls poorly when tapped down to the bottom of the vials. We quantified this behavior using a startle-induced climbing assay. Wild-type and heterozygous *Fer2* mutants showed similar climbing abilities at least during the first 3 weeks of the adult life. In contrast, all *Fer2* homozygous or hemizygous mutants displayed severely impaired climbing abilities throughout adulthood (Fig. 1C). We generated a driver fly line expressing *GAL4* under the control of the *Fer2* promoter (*Fer2-GAL4*) and a UAS line expressing FLAG-tagged *Fer2* cDNA (*UAS-Fer2-FLAG*). The expression of *Fer2-FLAG* with *Fer2-GAL4* partially but significantly rescued the decreased climbing ability of the *Fer2^1^* flies and restored the climbing ability of the *Fer2^2^* flies to the control level (Fig. 1D). These data indicate that *Fer2* is necessary for the startle-induced climbing ability.

### *Fer2* is required for the development and survival of dopaminergic neurons

**Figure 2:**
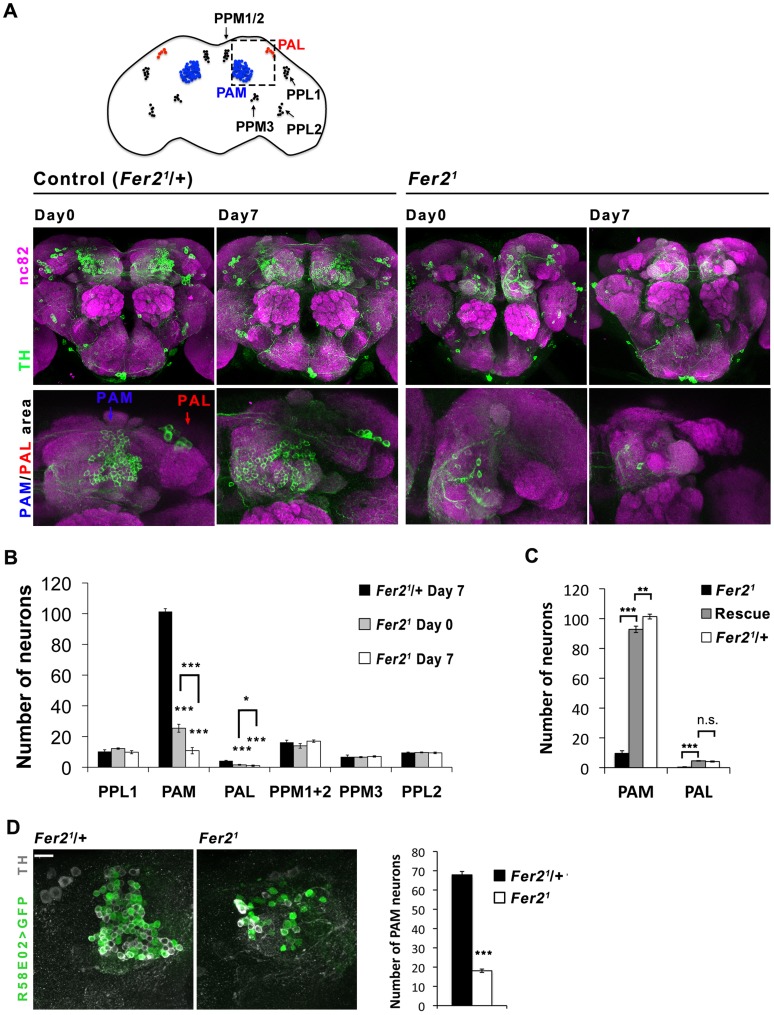
Selective loss of PAM and PAL cluster DA neurons in the *Fer2* extreme hypomorphic mutants. (A) Top, a diagram of DA neuron clusters in the adult fly brain. Bottom, representative images of the brains stained with anti-TH antibodies. Central brain and anterior brain region where PAM and PAL neurons are located are shown. (B) Quantification of DA neurons per hemisphere detected by TH-staining. For PAM and PAL neurons, *HL9 > GFP* and TH double positive neurons were quantified. *Fer2^1^/+*, n = 17. *Fer2^1^*: day 0, n = 12; day 7, n = 24. Mean ± SEM, * p<0.05, *** p<0.001, comparing *Fer2^1^* and *Fer2^1^/+* or *Fer2^1^* day 0 vs. day 7. No significant differences were found between *Fer2^1^* and *Fer2^1^/+* where no asterisks are shown. (C) Quantification of the PAM and PAL neurons in the 7-day-old *Fer2^1^* (n = 8), Rescue (*Fer2^1^*, *Fer2 > Fer2-FLAG*; n = 10) and control (*Fer2^1^/+*; n = 17) flies. (D) Left, representative confocal images of the brains of the 0-day-old flies expressing *UAS-GFP* with *R58E02-GAL4* double stained for GFP and TH. Right, quantification of the GFP/TH double positive neurons. Mean ± SEM, ***p<0.001. *Fer2^1^/+*, n = 7. *Fer2^1^*, n = 14.

We have previously shown that *Fer2^1^* mutation impairs the development of LNvs, which express the neuropeptide pigment-dispersing factor (PDF) [17]. Although it has been shown that PDF is necessary for the normal negative geotaxis behavior [23], whether PDF or LNvs are necessary for startle-induced climbing has not been documented. We found that the expression of *UAS-hid* with *Pdf-GAL4*, which selectively ablates LNvs [24], does not impair the startle-induced climbing ability (Fig. 1E, F). Thus, the decrease in startle-induced climbing ability in *Fer2* mutant flies is due to the deficits other than the lack of LNvs. Because loss of climbing ability is often associated with impaired CNS integrity [25] and the available transcriptome data indicate that *Fer2* is almost exclusively expressed in the brain (modENCODE Tissue Expression Data, FlyAtlas [26]), we examined the integrity of major neuron types in the brains of adult *Fer2^1^* mutants. We did not find any obvious differences in the overall morphology of the cholinergic, glutamatergic and serotonergic neurons between *Fer2^1^* and controls, although we cannot exclude the possibility that there are subtle differences in the number of these neurons (Fig. S1). Interestingly, we found an evident reduction of dopaminergic (DA) neurons in *Fer2^1^* mutants. Seven DA neuron clusters were detected by anti-tyrosine hydroxylase (TH) staining in the *Fer2^1^* heterozygouse flies, which were very similar in number and morphology to those in wild-type flies [27]. In contrast, there were markedly fewer DA neurons in the PAM and PAL clusters in homozygous *Fer2^1^* flies on the first day after eclosion (day 0); there were even fewer of them in 7-day-old flies (Fig. 2A). In addition, we expressed *UAS-GFP* under the control of the *HL9-GAL4* driver to label several clusters of DA neurons [28] and found a similar dramatic reduction of PAM and PAL neurons in the homozygous *Fer2^1^* flies (Fig. S2A). This indicates that PAM and PAL neurons were reduced in number in *Fer2^1^* mutants, rather than merely having reduced TH expression. Quantification of the *HL9 > GFP*-positive neurons revealed a 75% reduction in PAM neuron counts already at day 0 and a 90% reduction at day 7 in *Fer2^1^* compared to the heterozygous controls. Four out of 5 PAL neurons were undetectable at day 0 in the *Fer2^1^* flies, and most brains had no PAL neurons at day 7. The numbers of other DA neuron clusters were not different between *Fer2^1^* and controls at both ages (Fig. 2B). To further verify the loss of DA neurons in the *Fer2^1^* flies, we expressed GFP using the *R58E02*-*GAL4* driver, which is derived from the promoter of the dopamine transporter gene and expressed almost exclusively in PAM neurons [29]. There were significantly fewer *R58E02*-*GAL4*-labeled PAM neurons in the *Fer2^1^* flies compared to the control, supporting the finding that a large fraction of PAM neurons were absent in *Fer2^1^* (Fig. 2D). The expression of *Fer2-FLAG* by *Fer2-GAL4* restored the loss of PAM and PAL neurons in the *Fer2^1^* flies to quasi wild-type levels (Fig. 2C).

**Figure 3:**
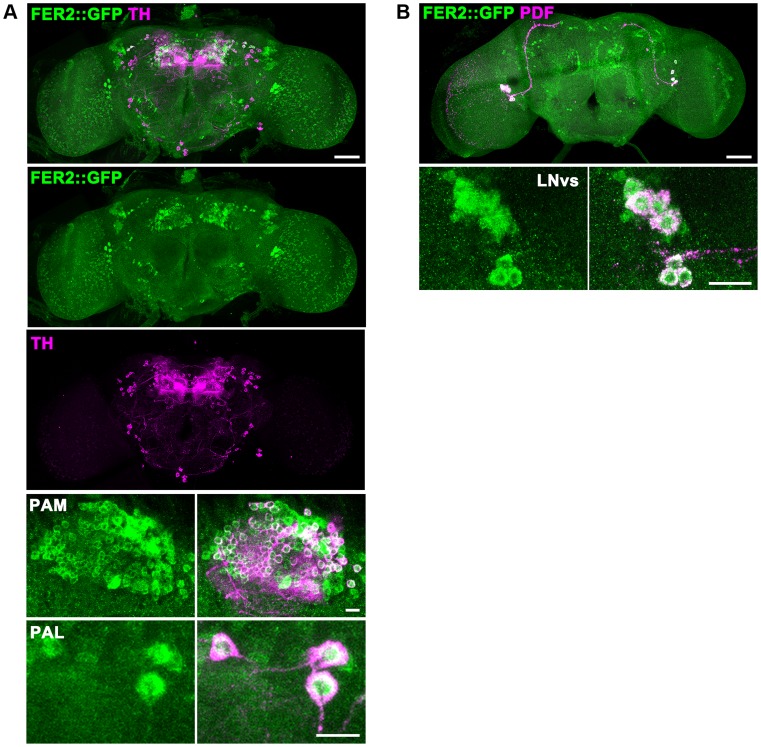
FER2 expression in the brain. FER2 is expressed in the PAM and PAL DA neurons and the LNvs. Brains of the *Fer2::GFP* flies were stained for GFP and TH (A) or GFP and PDF (B). Scale bars in the whole brain images are 50 µm, 10 µm in the insets.

To examine if *Fer2* is expressed in PAM and PAL neurons, we monitored the expression of a GFP-tagged *Fer2* genomic transgene in the brain by GFP/TH double staining. FER2::GFP expression was observed in all the PAM and PAL neurons and in a few other clusters of cells. As expected, GFP/PDF double staining confirmed the expression of FER2::GFP in the LNvs, consistent with the previous RNA analysis results [17] (Fig. 3A, B). *Fer2*-*GAL4* showed a more widespread expression pattern than FER2::GFP, as is often the case with promoter-GAL4s. Nonetheless, *Fer2*-*GAL4* was also expressed in all PAM neurons and 4 out of 5 PAL neurons (Fig. S2B). Having validated the expression of *Fer2-GAL4* in PAM and PAL neurons, we next used it to express *UAS-TH* in the *Fer2^1^* flies and immunostained the brains with anti-TH antibodies. *Fer2* > *TH* slightly increased the number of neurons detected by TH-staining in the PAM and PAL clusters but not to the control level, which demonstrates again the absence of these cells (Fig. S2C). These results suggest that *Fer2* is expressed in PAM and PAL neurons and further support that *Fer2^1^* mutation selectively reduces the number of these neurons.

To examine the possibility that the dopaminergic neurotransmitter identity of PAM and PAL neurons is changed in *Fer2^1^* and thus they are undetectable, we analyzed the cell lineage derived from the *Fer2-GAL4*-expressing cells. By combining *Fer2-GAL4, UAS-FLP* and *Ubi^P63^ > stop> EGFP*, the *Fer2-GAL4*-expressing lineage was marked with GFP in the control and *Fer2^1^* flies (Fig. S2D) [30]. While most of the PAM and 4 PAL neurons were GFP-positive in the heterozygous control flies, the majority of these neurons were not present and no ectopic GFP-positive cells were observed in *Fer2^1^* (Fig. S2E, F). Therefore, the reduction of PAM and PAL neurons in *Fer2* ^1^ is not due to a cell-fate switch.

To learn more about the developmental impairments of PAM and PAL neurons in *Fer2^1^* mutants, we next examined DA neurons in the pupal brains with anti-TH staining, because these neurons are not present in the larval brain and some of the PAM neurons are known to be born during pupal stages [27], [31]. In the *Fer2^1^* heterozygous controls, approximately 80% of PAM and PAL neurons were clearly detectable within 5 days after puparium formation (APF). Whereas in *Fer2^1^* homozygotes, PAM and PAL neurons gradually increased in number but were significantly fewer than in the controls throughout pupal development. These observations indicate that the majority of PAM and PAL neurons were not formed or died before maturation into DA neurons in *Fer2^1^* mutants (Fig. S3A, B). Taken together with the observation that the loss of PAM/PAL neurons progresses at least up to 7 days into adulthood (Fig. 2B), these results indicate that *Fer2^1^* mutation impairs the development of the DA neurons in the PAM and PAL clusters and also causes their rapid degeneration in adulthood.

### *Fer2* is a survival factor for PAM neurons in aging flies

**Figure 4:**
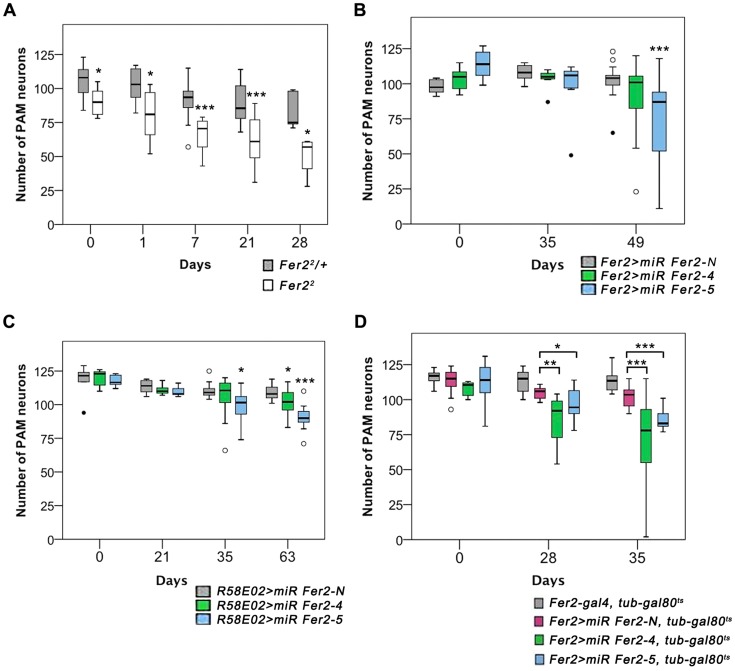
Partial loss of *Fer2* expression impairs the viability of PAM neurons in aging flies. Quantification of PAM neurons in the flies of different genotypes detected by anti-TH staining at indicated ages. Box boundaries are the 25th and 75th percentiles, the horizontal line across the box is the median, and the whiskers are the lowest and highest values that are not outliers. The open circles represent outliers and closed circles are extreme cases. *p<0.05, **p<0.01, *** p<0.001, comparing *Fer2^2^/+* and *Fer2^2^* at the same age (A), or between *miR-Fer2-N* and *miR-Fer2-4/*-*5* at the same age (B-D). Sample sizes are indicated in the Materials and Methods. (A) PAM neurons in *Fer2^2^* and the control (*Fer2^2^/+*). (B) Constitutive expression of *miR Fer2s* by *Fer2-GAL4.* (C) Constitutive expression of *miR Fer2s* by *R58E02-GAL4*. (D) Adult-specific *Fer2* knockdown and the control. *Fer2-GAL4*, *tub-gal80^ts^*: control without UAS-transgene.

We next asked whether the integrity of DA neurons is affected in the milder hypomorphic mutant *Fer2^2^* as well. We focused our analysis on the PAM cluster DA neurons and monitored their number in the *Fer2^2^* flies by anti-TH staining. At day 0, the number of PAM neurons was slightly reduced in *Fer2^2^* compared to the control, but to a much lesser extent as in *Fer2^1^*. Remarkably, the loss of PAM neurons continued progressively at least up to 28 days in *Fer2^2^* (Fig. 4A). Lineage tracing using *HL9-GAL4, UAS-FLP* and *Ubi^P63^ > stop > EGFP* flies verified the loss of PAM neurons in *Fer2^2^* (Fig. S4A). The loss of PAM neurons was rescued by expressing *Fer2-FLAG* with *Fer2-GAL4* (Fig. S4B).

The contrasting results between *Fer2^1^* and *Fer2^2^* mutants suggest that a moderate reduction of *Fer2* expression has only a minor effect on the development of DA neurons but is sufficient to deteriorate PAM neurons in aged flies. To test this, we generated UAS-transgenic lines to express 2 independent microRNAs (miRNAs) that target *Fer2* (*miR Fer2-4* and *-5*) and one negative control miRNA (*miR Fer2-N*) that contains a sequence of 19 random nucleotides unrelated to any *Drosophila* gene (see Fig. 1A). When expressed with *Fer2-GAL4*, both *miR Fer2-4* and *-5* reduced the *Fer2* mRNA levels to approximately 35% of the wild-type level, whereas *miR Fer2-N* had no effect on the *Fer2* mRNA (Fig. S5A). As expected, constitutive expression of *miR Fer2-N* by *Fer2-GAL4* at 25°C did not alter the number of PAM neurons. In the flies expressing *miR Fer2-4* or *miR Fer2-5*, the number of PAM neurons remained stable until 35 days of age. However, at 49 days, many flies in either of the knockdowns had a reduced number of PAM neurons, and the reduction was significant in *Fer2 > miR Fer2-5* flies (Fig. 4B). The constitutive knockdown by *miR Fer2-4* or *miR Fer2-5* at 25°C with *R58E02-GAL4* or *HL9-GAL4* resulted in a similar trend, with a significant reduction of PAM neurons in the flies aged over several weeks old (Fig. 4C, S5B). The PAL and other DA neuron clusters were not affected by any of the *Fer2* knockdowns, although *Fer2-GAL4* and *HL9-GAL4* are expressed in most of the DA clusters including PAL neurons. While there were subtle differences in the onset of degeneration that were likely due to the differences in GAL4 expression levels, the data nevertheless illustrate that *Fer2* knockdown causes adult-onset PAM neuron degeneration.

The foregoing observations indicate that a moderate reduction of *Fer2* expression either by a hypomorphic mutation or by knockdown has little effect on DA neuron development but mainly affects the survival of PAM neurons in adults. This further suggests that the role of *Fer2* in the survival of adult PAM neurons is independent of its role in development. To test this more directly, we knocked-down *Fer2* only during adulthood using a combination of *UAS-miR Fer2s*, *Fer2-GAL4* and temperature-sensitive GAL80 expressed under the *tubulin* promoter (*tub-GAL80^ts^*). These flies were reared at 18°C (a permissive temperature for GAL80^ts^) until eclosion, and then the temperature was shifted to 29°C (a restrictive temperature for *GAL80^ts^*) to allow for transcriptional activation by GAL4 throughout adulthood [32]. We found that the adult-specific knockdown of *Fer2* induced the adult-onset progressive degeneration of PAM neurons without affecting PAL neurons (Fig. 4D). Notably, the loss of PAM neurons was more evident in these flies than in flies with constitutive knockdown (Fig. 4B), which is consistent with the greater GAL4 activity at 29°C than at 25°C [33]. These results clearly distinguish the role of *Fer2* in developing and adult DA neurons and demonstrate that *Fer2* expression is required for the survival of adult PAM neurons in aging flies.

### Loss of PAM neurons leads to the locomotor deficits

**Figure 5:**
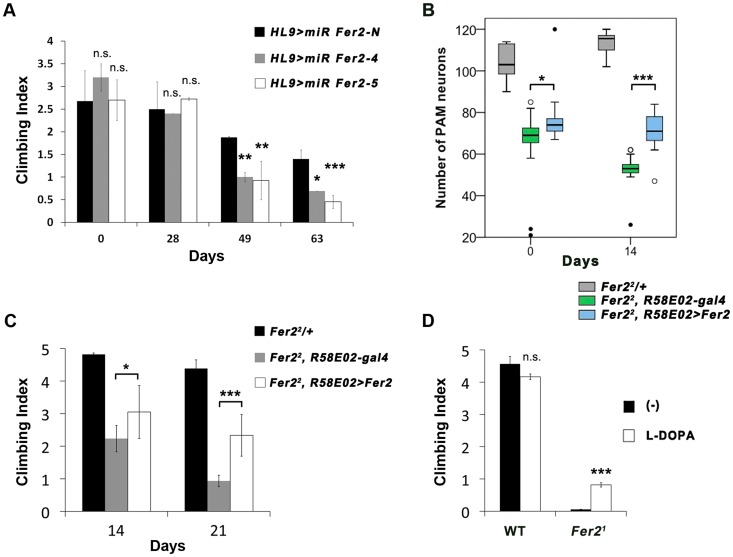
Loss of PAM neurons leads to the locomotor deficits. (A) Climbing abilities of the flies expressing *Fer2* miRNAs or negative control miRNA with *HL9-GAL4*. Mean from 3 independent experiments ± SEM. * p<0.05, ** p<0.01, ***p<0.001. (B and C) PAM neuron-targeted expression of *Fer2-FLAG* by *R58E02-GAL4* significantly rescued the degeneration of PAM neurons and climbing ability in the *Fer2^2^* mutants. Sample sizes are in the Materials and Methods. (B) PAM neuron counts. *Fer2^2^/+*: positive control. *Fer2^2^*, *R58E02-gal4*: negative control. *Fer2^2^*, *R58E02 > Fer2*: rescue. * p<0.05, *** p<0.001. (C) Mean Climbing Index from 3 independent experiments ± SEM. * p<0.05, *** p<0.001. (D) Climbing ability of the 3-day-old wild-type (*WT*) and *Fer2^1^* mutant flies fed with or without L-dopa. Mean ± SEM. ***p<0.001.

We next asked whether the loss of PAM neurons, which is the most prominent cellular phenotype in *Fer2* mutants, is the cause of their locomotor impairment. To test the role of PAM neurons in the startle-induced climbing ability more directly, we knocked-down *Fer2* in PAM neurons by *HL9-GAL4* and performed a climbing assay. Knocking-down *Fer2* with either of the miRNAs resulted in significant declines in climbing ability after 49 days (Fig. 5A). This is consistent with the observation that *HL9 > miR Fer2s* induce the adult-onset degeneration of PAM neurons only after several weeks (Fig. S5B).

To further assess the contribution of PAM neurons in the climbing ability, we sought to rescue the loss of PAM neurons using a PAM neuron-specific driver in *Fer2* mutant flies. Expression of *UAS-Fer2-FLAG* using *HL9-GAL4* or *R58E02-GAL4* did not rescue the loss of PAM neurons in *Fer2^1^*. This is most likely because the majority of PAM neurons fail to form or die in *Fer2^1^* before these DA neuron-specific drivers start to be expressed, consistent with the fact that *R58E02-GAL4* has little expression in the larval brain (H. Tanimoto and A. Thum, personal communication). Thus, we reasoned that PAM-neuron specific rescue might be possible in *Fer2^2^* flies, which show little impairments in DA neuron development but display progressive PAM neuron degeneration. Indeed, *R58E02 > Fer2-FLAG* suppressed the degeneration of PAM neurons in the *Fer2^2^* mutants in adulthood (Fig. 5B). A climbing assay revealed that *R58E02 > Fer2-FLAG* significantly improves the climbing impairments in the *Fer2^2^* flies (Fig. 5C). The rescue of the climbing ability was partial. This may be because some of the PAM neurons that failed to develop in *Fer2^2^* were not rescued, or because other unknown cell types affected in *Fer2^2^* contribute to the climbing ability. Nevertheless, these observations together with the results of the PAM neuron targeted-knockdown indicate that PAM neurons are necessary, although may not be sufficient, for the normal climbing ability of the flies.

The dopaminergic system is critically involved in the control of locomotion in both vertebrates and invertebrates [34], [35]. The motor symptoms of PD arise mainly from the loss of DA neurons in the SNc [5]. L-dopa, a dopamine biosynthesis precursor, remains the gold standard for treatments of PD motor symptoms. We found that the locomotor deficit of the *Fer2^1^* flies was partially but significantly rescued by feeding with L-dopa (Fig. 5D). By anti-TH staining, we observed no significant rescue of the number of DA neurons by L-dopa, which is consistent with a previous study [36]. Since L-dopa has to be converted to dopamine in the DA neuron terminals to exert its therapeutic effect, the partial rescue of the locomotion by L-dopa is also consistent with the marked loss of PAM neurons observed in *Fer2^1^* mutants.

### *Fer2* plays a critical role in oxidative stress response and protection of PAM neurons against oxidative insults

**Figure 6:**
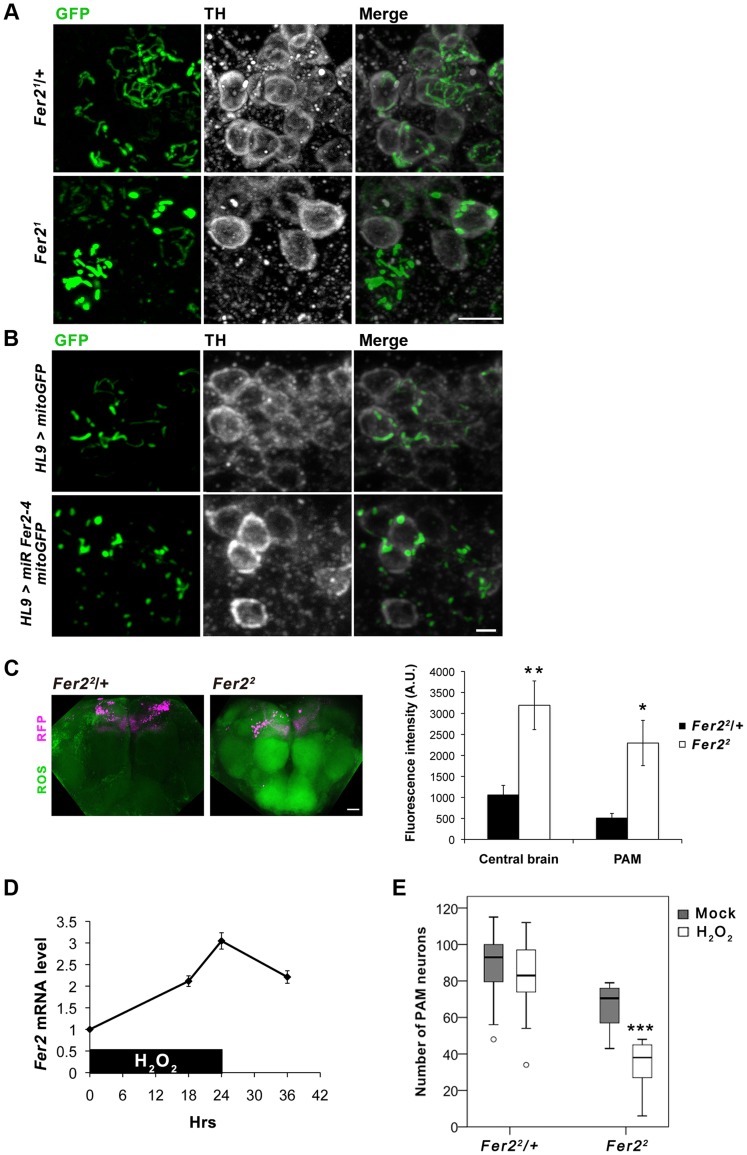
*Fer2* protects PAM neurons from oxidative stress. (A-B) Mitochondrial morphology in PAM neurons visualized by expressing *mitoGFP* with *HL9-GAL4* and double stained for GFP and TH. (A) 1-day-old control (*Fer2^1^/+*) and *Fer2^1^* mutant flies. Scale bar, 5 µm. (B) DA neuron-targeted *Fer2* knockdown by *HL9 > miR Fer2-4* and control (14-day-old). Scale bar, 2 µm. (C) ROS levels in the 5-day-old control (*Fer2^2^/+*) and *Fer2^2^* flies monitored using H2DCF dye. Left, representative confocal images of the ROS production in the brain. PAM neurons were RFP-labeled with *R58E02-GAL4*. Scale bar, 30 µm. Right, quantification of the ROS levels. Mean ± SEM. Control, n = 5. *Fer2^2^*, n = 5. Two independent experiments were performed with similar results. * p<0.05, ** p<0.01. (D) Relative *Fer2* mRNA levels in wild-type flies during and after 24-hr 5% H_2_O_2_ treatment (5-day-old at the onset of the treatment). Data are shown as *Fer2* mRNA levels of the H_2_O_2_-treated flies relative to the mock-treated controls. Mean ± SEM from 2–3 independent experiments. (E) PAM neuron counts in the *Fer2^2^* heterozygous and homozygous flies with or without H_2_O_2_ treatment. *Fer2^2^/+*: control, n = 40; H_2_O_2_, n = 14. *Fer2^2^*: control, n = 10; H_2_O_2_, n = 14. *** p<0.001.

Accumulating evidence suggests that dysfunctions in multiple aspects of mitochondrial biology are associated with the DA neurodegeneration in PD and pathogenesis of other neurodegenerative disorders [37], [38]. To examine whether mitochondrial dysfunction is involved in the loss of DA neurons caused by the loss of *Fer2* expression, we visualized mitochondria in the adult PAM neurons by expressing mitochondria-targeted GFP (mitoGFP) [39] with *HL9*-*GAL4*. The majority of visible mitochondria in the cell bodies of the remaining PAM neurons in *Fer2^1^* mutants was in enlarged aggregations and did not form tubular networks as in the control flies (Fig. 6A). Similarly, DA neuron-selective *Fer2* knockdown by *HL9-GAL4* and *Fer2^2^* mutation lead to the accumulation of abnormally enlarged mitochondria in PAM neurons (Fig. 6B, S6A). Mitochondrial morphology in some of the *TRH-GAL4*-positive serotonergic neurons was indistinguishable between *Fer2^1^* homozygotes and heterozygotes, suggesting that mitochondria in PAM neurons are particularly vulnerable to loss of *Fer2* expression (Fig. S6B).

Since mitochondria are the major source of ROS, mitochondrial dysfunction leads to an excessive ROS production and oxidative damages to various macromolecules. Oxidative stress causes rapid depolarization of mitochondrial inner membrane and inhibits complex I activity, exacerbating ROS production. Thus, mitochondrial defects and elevated ROS levels are interdependent and are thought to have prominent roles in PD pathogenesis [40]. We therefore asked whether ROS levels are increased in the *Fer2* mutant brains and if it has a causative role on PAM neuron degeneration. We monitored intracellular ROS levels in the brains of *Fer2^2^* mutant and the heterozygous control flies using 2′,7′-dichlorofluorescein (H2DCF), which produces green fluorescence upon reacting with ROS. Interestingly, ROS levels were significantly elevated throughout the brain in 5-day-old *Fer2^2^* flies compared with the age-matched controls. Although there was no regional specificity of ROS accumulation, ROS levels within the PAM neurons were also significantly elevated in *Fer2^2^* (Fig. 6C). These suggest that loss of *Fer2* expression leads to a systemic increase in oxidative stress in the brain.

The surprisingly dramatic increase of ROS levels in *Fer2^2^* mutants prompted us to further examine if *Fer2* is involved in oxidative stress response. We first tested if *Fer2* expression levels can be altered upon oxidative stress-challenge by feeding flies with non-lethal dose of hydrogen peroxide (H_2_O_2_, 5%) for 24 hr. We found that H_2_O_2_ treatment significantly increases *Fer2* mRNA levels (Fig. 6D). We next examined whether oxidative stress-challenge aggravates the degeneration of PAM neurons in *Fer2* mutants by anti-TH staining. The H_2_O_2_ treatment did not affect PAM neurons in *Fer2* ^2^ heterozygous flies, whereas the number of PAM neurons was significantly decreased in *Fer2* ^2^ homozygotes after the treatment (Fig. 6E). DA neuron counts in other clusters were unchanged by the same H_2_O_2_ treatment in *Fer2^2^* mutants (Fig, S6C), indicating that loss of *Fer2* expression renders PAM neurons selectively more vulnerable to increased oxidative stress. Taken together, these results point toward a role for *Fer2* in oxidative stress response and suggest that *Fer2* contributes to the protection of PAM neurons against oxidative stress.

### *C.elegans hlh-13* is required for the survival of DA neurons under oxidative stress

**Figure 7:**
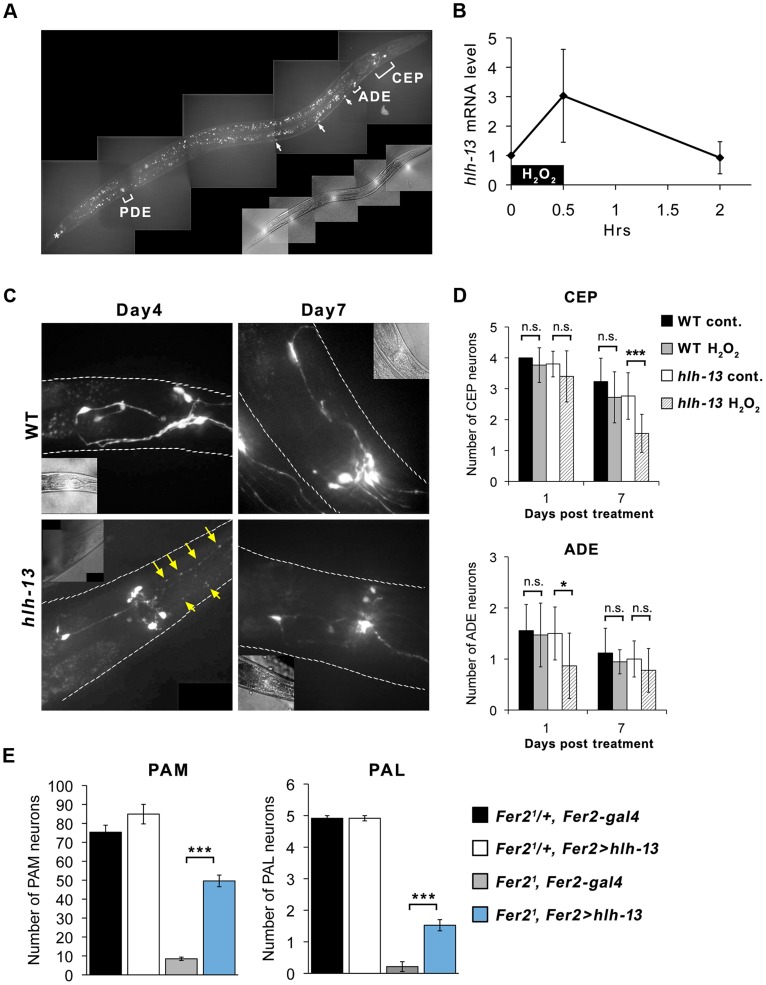
*C.elegans hlh-13* and *Drosophila Fer2* share a role in DA neuron protection under oxidative stress. (A) *GFP::hlh-13* is expressed in DA neurons (CEP, ADE and PDE), unidentified ventral nerve cells (arrows), and in a tail neuron (asterisk). Fluorescence signal that is not labeled is the gut autofluorescence. A representative image of a L4 worm. (B) *hlh-13 mRNA* levels in wild-type worms after a brief 1 mM H_2_O_2_ treatment quantified by qPCR relative to the mock-treatment value. Mean from 4 independent experiments. Error bar, SEM. (C) Representative images of GFP-labeled DA neurons in the head in wild-type and *hlh-13(tm2279)* worms after H_2_O_2_ treatment. Arrows indicate fragmented CEP neuron projections. (D) Quantification of CEP and ADE neurons in the H_2_O_2_-treated and non-treated worms. Mean ± SEM. * p<0.05, *** p<0.001. Between 14 and 18 worms were examined for each condition. (E) Loss of PAM and PAL neurons in the *Fer2^1^* flies was significantly rescued by expressing *hlh-13* with *Fer2-GAL4*. *Fer2^1^/+*, *Fer2-gal4*: n = 13. *Fer2^1^/+*, *Fer2 > hlh-13*: n = 13. *Fer2^2^*, *Fer2-gal4*: n = 16. *Fer2^1^*, *Fer2 > hlh-13*: n = 22. 1-day-old flies. Mean ± SEM. *** p<0.001.

*Fer2* homologs are found from nematodes to vertebrates [41]. *hlh-13* is predicted to be the single homolog of *Drosophila Fer2* and mammalian *p48/ptf1a* in *C.elegans* [42]. Consistent with a previous study [42], a *GFP::hlh-13* genomic transgene was expressed in all DA neurons (named CEP (4 cells), ADE (2 cells) and PDE (2 cells)) and in a tail neuron in developing and adult worms. *GFP::hlh-13* expression was also observed in several unidentified ventral nerve cells from L2 to L4 stages (Fig. 7A).

Since both fly *Fer2* and worm *hlh-13* are expressed in DA neurons, we next asked whether *hlh-13* has a comparable function as *Fer2* in DA neuron development or survival. We used the *hlh-13* knockout mutant *hlh-13*(*tm2279*) to test the effect of *hlh-13* loss-of-function on the number of DA neurons and on a dopamine-dependent behavior, the basal slowing response. The basal slowing response is a slowing of locomotion rate when worms encounter bacteria and has been shown to require dopamine signaling [43] (Text S1). The knockout mutant showed no differences in the number of DA neurons and basal slowing response compared to wild-type worms (Fig. 7D control, Fig. S7A). Therefore, unlike *Fer2*, *hlh-13* is not required for the development, survival or function of DA neurons under normal growth conditions.

Next, to test if *hlh-13* is involved in the survival of DA neurons under oxidative stress, we treated wild-type and *hlh-13*(*tm2279*) mutant adult worms with 1 mM H_2_O_2_ for 30 min and analyzed the *hlh-13* mRNA levels and DA neuron integrity at subsequent time points. *hlh-13* mRNA levels were upregulated by approximately 3-fold immediately after the H_2_O_2_ treatment and returned to the non-treated levels after 2 hrs (Fig. 7B). To monitor DA neurons in the wild-type or *hlh-13*(*tm2279*) background, we used the *dat-1::gfp* reporter driving GFP expression in DA neurons. Since it was difficult to reliably detect PDE neurons, we focused our analysis on CEP and ADE neurons located in the head. In wild-type animals, at least until 7 days after the H_2_O_2_ treatment, there were no significant differences in the number or morphology of the CEP and ADE neurons between treated and untreated groups. By contrast, H_2_O_2_- treated *hlh-13(tm2279)* mutants showed fragmentation of the CEP neuron projections starting from day 4 after treatment, followed by the loss of cell bodies. Similarly, the number of ADE neurons was also reduced in the H_2_O_2_-treated mutants (Fig. 7C, D). Despite the apparent change in DA neuron numbers, the basal slowing response was not different between the control and stressed worms in either genotype (Fig. S7B), which is consistent with the previous observation that basal slowing response is defective only when all 4 CEPs are ablated [43]. These results indicate that, similar to fly *Fer2*, *hlh-13* is likely to be involved in the oxidative stress response and required for the protection of DA neurons under oxidative stress in adult worms.

To examine the extent to which *hlh-13* shares the function with *Fer2*, we next sought to test cross-species complementation of the *Fer2* loss-of-function mutation in flies by the *hlh-13* gene. We generated a *UAS-hlh-13* construct and expressed it with *Fer2-GAL4* in the *Fer2^1^* mutant background. We found that the loss of PAM and PAL neurons in *Fer2^1^* was partially but significantly rescued by the expression of *hlh-13* (Fig. 7E). Collectively, these results confirm that *hlh-13* is the *C.elegans* ortholog of *Fer2* and suggest that the protection of DA neurons against oxidative insults is a conserved role between these orthologs.

## Discussion

Many neurodegenerative disorders are multi-factorial, in which interactions between environmental and genetic factors play important causal roles. Oxidative stress has emerged as a major pathogenic factor for common neurodegenerative diseases, yet how such a ubiquitous phenomenon leads to the loss of selective neuronal populations remains unclear [44]. Here we presented evidence that loss-of-function in *p48* homologs in *Drosophila* and *C.elegans* renders DA neurons susceptible to degeneration under oxidative stress in adult animals. Interestingly, genome-wide association studies for PD have identified candidate causal SNPs in *p48/ptf1a* [7], [16], suggesting the possibility that *p48* loss-of-function may represent an as-yet-unknown genetic risk factor that increases susceptibility of DA neurons to environmental toxins also in mammals. Many familial PD-associated genes are widely expressed; nevertheless, mutations in these genes result in a selective loss of SNc DA neurons, suggesting that cell-type-specific factors, those similar to *Fer2* and *hlh-13*, might contribute to the DA neuron vulnerability even in the familial PD cases. The identification of *Fer2* and *hlh-13* upstream and downstream pathways may thus shed light on the common mechanisms underlying the selective loss of DA neurons in diverse PD cases.

The major cellular phenotype in *Fer2^1^* mutants was the developmental defects in 2 subsets of DA neurons, in addition to the developmental loss of LNvs [17], although we cannot exclude the possibility that other neuronal types are also affected (Fig 2, S1). Judging from the results of the lineage-tracing experiments and the observation of DA neurons in the pupal brain, *Fer2* is not a selector gene for dopaminergic phenotype in PAM/PAL neurons but is required for neurogenesis or survival of postmitotic neurons before phenotypic maturation (Fig. S2E, F and S3). The notion that genes required for the development of DA neurons confer important roles in adult DA neuron survival has been postulated by several studies in mammals [8], [9], [10], [11]. Although the molecular mechanisms underlying their roles in adult neurons remain elusive, these developmental genes may actively control the genetic programs required for the maintenance of cell identity in adults [45]. Our findings on the *Fer2*'s dual roles extend this notion to invertebrate nervous systems and underscore its significance.

PAM neuron-targeted *Fer2* knockdown induces PAM neuron degeneration (Fig. 4C) and mitochondrial dysfunction within PAM neurons (Fig. 6B, S6A). These results indicate that mitochondrial dysfunction and cell death can be induced by a cell-autonomous reduction of *Fer2* expression within the PAM cluster. On the other hand, ROS levels are increased brain-wide in the *Fer2^2^* flies, despite the fact that *Fer2* expression is restricted to several clusters of cells in the brain (Fig. 3, 6C). Thus, loss of *Fer2* expression leads to both cell-autonomous and non-cell-autonomous consequences to the animal's well-being. How does the brain-wide ROS increase occur by *Fer2* mutation although *Fer2* is not expressed ubiquitously? An intriguing recent study in *C. elegans* demonstrated that mitochondrial perturbation in neuronal cells modulates mitochondrial stress response in distal tissues non-cell-autonomously [46]. Flies might exhibit similar non-cell-autonomous mitochondrial stress response that causes systemic ROS production. Systemic increase in oxidative stress is a clinical feature common to many aging-related neurological diseases including PD [47]. Studies in mammals have documented that inflammation is a major factor mediating excessive ROS production and PD pathology. Activated microglia produces ROS and mediates DA neuron death. Dying DA neurons stimulate microglia, exacerbating the ROS production and DA neurodegeneration [48]. As CNS glia in *Drosophila* are thought to possess immune-like function [49], similar mechanisms via inflammatory responses might mediate global elevation of ROS production in *Fer2* mutants.

**Figure 8:**
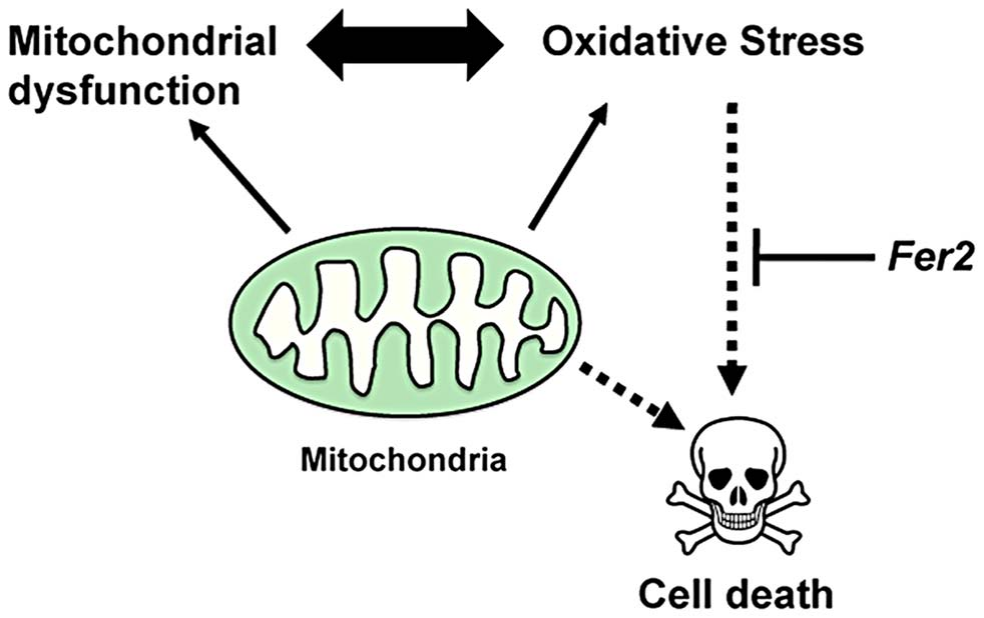
A model for the role of *Fer2* in the survival of DA neurons. Mitochondrial dysfunction and oxidative stress are interdependent and are both known to mediate DA neuron death. *Fer2* expression is upregulated in response to oxidative stress and counteracts PAM neuron degeneration.

Are the abnormal mitochondria in PAM neurons a cause or a consequence of the ROS upregulation? Because mitochondrial defects and excessive ROS production are inter-dependent, it is not possible to clarify the causality in the current study. However, because *Fer2* expression is upregulated upon H_2_O_2_ treatment and the same acute H_2_O_2_ treatment triggers PAM neuron death in the absence of *Fer2* (Fig. 6D, E), we favor the hypothesis that *Fer2* provides protection against oxidative stress rather than directly acting on mitochondria (Fig. 8). These phenomena are remarkably similar in *C.elegans*; an acute H_2_O_2_ treatment upregulates *hlh-13* expression and triggers DA neuron degeneration in *hlh-13* null mutants (Fig. 7B–D). These data suggest that the oxidative stress response is an ancestral role of *p48* homologs. Alternatively, *hlh-13*'s roles in neural development in worms might have been taken over by other genes. Either way, these findings suggest that loss-of-function in *Fer2* and *hlh-13* can be used to study pathophysiology of DA neuron degeneration under oxidative stress. Interestingly, *Fer2* mRNA levels remain upregulated at least up to 12 hr after the 24-hr H_2_O_2_ treatment, whereas *hlh-13* mRNA levels return to the non-treated levels 2 hr after a brief H_2_O_2_ treatment (Fig. 6D, 7B). This difference in gene expression kinetics may reflect the duration of the H_2_O_2_ treatment, RNA stability, or difference in signal transduction mechanisms. Various stress response genes show highly restricted temporal expression upon stress, as the continuous activation of these genes are often detrimental to the cell [50]. Initial upregulation of *hlh-13* immediately after an acute oxidative stress might be necessary and sufficient to trigger the downstream genetic programs that continue to scavenge ROS and repair the cellular damages during the following days. Identification of the downstream genetic programs controlled by *Fer2* and *hlh-13* will be a key toward understanding the evolutionarily conserved mechanisms of neuroprotection.

Mild loss of *Fer2* expression by *Fer2^2^* mutation or knockdown leads to a progressive loss of PAM neurons associated with mitochondrial dysfunction, increase in ROS production and progressive locomotor deficits, all of which are reminiscent of the pathological characteristics of PD. Unlike other fly PD models that are derived from genetic modifications of human PD-associated genes or their homologs, *Fer2* is not an ortholog of known familial PD-associated genes. Yet, the magnitude of the DA neuron degeneration caused by the loss of *Fer2* expression is markedly greater than in existing fly PD models [51]. We demonstrated that the loss of PAM neurons is at least partly responsible for the impaired climbing ability caused by *Fer2* loss-of-function (Fig 5A–C). Because rescue of the PAM neuron counts in *Fer2^1^* mutants to quasi wild-type level does not restore the climbing ability to the control level (compare Fig. 1D and 2C), it is likely that some cells other than PAM and PAL neurons are somehow affected in *Fer2^1^* and contribute to the locomotor deficits. Nonetheless, our results are in agreement with the recent study by S. Birman and colleagues, which demonstrates that the progressive motor deficits in the flies expressing human *α-synuclein*, a transgenic model of PD, derives from the dysfunction of a subset of PAM neurons [52]. Because the selective degeneration of DA neurons within the SNc is the principal cause of the motor manifestations of PD, the *Drosophila* PAM neurons parallel the DA neurons of the human SNc with regard to function and vulnerability. Thus, *Fer2* loss-of-function may serve as a model to better understand the mechanisms by which the loss of specific subsets of DA neurons leads to locomotor deficits in PD.

## Materials and Methods

### Fly strains

*PBac {RB} Fer2^e03248^* (*Fer2^1^*) has been previously characterized [17]. The following lines were obtained from the Bloomington Stock Center: *Df(3R)Exel7328* (referred to herein as *Df*), *Mi{ET1}Fer2^MB09480^* (*Fer2^2^*), *UAS-mitoGFP* and the strain used for *Fer2-GAL4* flip-out assay (*w; P{UAS-RedStinger}4, P{UAS-FLP1.D}JD1, P{Ubi-p63E(FRT.STOP)Stinger}9F6/CyO*) [30]. *HL9*-*GAL4* [28] was a gift from G. Miesenböck. *dvglut ^CNSIII^-GAL4* and *Cha-GAL4* [53] were gifts from A. DiAntonio. *TRH-GAL4* [54] was from O. Alekseyenko. *DILP2-GAL4* [55] was a gift from P. Léopold. *R58E02-GAL4* [29] was from H.Tanimoto and *UAS-TH* [56] was from J.True. *Fer2::GFP* genomic transgene FlyFos022529 was derived from a fosmid clone including approximately 5 kb upstream and 20 kb downstream of the *Fer2* gene. FlyFos022529 (pRedFlp-Hgr) (Fer2[16092]::2XTY1-SGFP-V5-preTEV-BLRP-3XFLAG)dFRT) was generated and generously provided by the project “A reverse genetic toolkit for systematic study of gene function and protein localization in Drosophila” (M.IF.A.MOZG8070).

To generate *Fer2-GAL4*, 2359 bp upstream of Fer2 ATG were amplified using the following primers: EagFer2upF, 5′- TTTCGGCCGTGGATTTGCTCTGGTTTGGATGC -3′ and XhoFer2upR, 5′- TTTCTCGAGTTTTACGCACTTCCGCTGTCC -3′. The amplified fragment was cloned into a pENTR 3c gateway vector (Invitrogen), verified by sequencing and cloned into to the transformation vector containing GAL4 (pBPGal4.2 Uw2; Addgene) using the gateway system (Invitrogen). The *Fer2-GAL4* transgene was inserted into the attP16 landing site [57] on the second chromosome by PhiC31-mediated recombination by a commercial transformation service (BestGene, Inc.). UAS-miR Fer2 constructs were generated as described in [58]. Four different miRNA coding sequences were generated along with a negative control, which does not target any sequence in the *Drosophila melanogaster* genome. From these 4, 2 miRNAs (lines 4 and 5) were used for further experiments. The miRNA sequence of line 4 was 5′-TGAGCAAGATCGACACTCTGC-3′, the miRNA sequence of line 5 was 5′-TCAAAGCGGATAGGGCTAATT-3′ and the sequence of the negative control miRNA was 5′- TACCCGTATCGGGTTAATCGA -3′. The stem loop structure was predicted using the RNAfold Webserver of the institute of Theoretical Chemistry at the University of Vienna (http://rna.tbi.univie.ac.at/cgi-bin/RNAfold.cgi). UAS-miR Fer2 constructs were inserted into the attP40 landing site on the second chromosome (BestGene, Inc.). To generate the UAS-Fer2-FLAG construct, *Fer2* cDNA with a 3xFLAG tag coding sequence at its 3′ end was amplified and cloned into pCR II Topo vector (Invitrogen) and verified by sequencing. A DNA fragment containing Fer2-FLAG coding sequence was then cloned into a UAS-containing transformation vector (pUAST-UAS-Stringer attB). The UAS-Fer2-FLAG construct was integrated into the attP40 landing site. The UAS-hlh-13 construct was generated by cloning a PCR-amplified hlh-13 full-length cDNA into the pBid-UASC-G vector [59] by Gateway cloning, and integrated into the attP40 landing site.

Throughout the text and in the figures, genotype “X>Y” indicates a combination of GAL4 (X) and UAS-effector (Y).

### Worm strains

*C.elegans* were cultured using standard protocol unless otherwise indicated. The following strains were obtained from the Caenorhabditis Genetic Center: wild-type (N2), BZ555 *egIs1[dat-1::gfp]* and IU189 *rwls1[hlh-13p::GFP::hlh-13,mec-7::RFP]*. IU129 *hlh-13(tm2279)* was a gift from S. Lee [42].

### Climbing assay

To assay the startle-induced locomotion of the flies, we used a negative geotaxis assay with modifications [60]. Twenty flies were anesthetized with CO_2_ and placed in a vertical glass column (25 cm length, 1.5 cm diameter) with a conical bottom. The columns were divided into 5 equally spaced zones and graded from 1 to 5 from the bottom to the top. After a 1-hr recovery period from CO_2_ exposure, the flies were gently tapped to the bottom. The flies were then allowed to climb the wall for the subsequent 20 seconds. The experiments were video recorded and the videos were manually analyzed using VLC software. The numbers of flies that climbed up to each zone within 20 seconds were counted. Flies that remained at the bottom were defined to be in zone 0. A climbing index (CI) was calculated using the following formula: CI =  (0×n_0_ + 1×n_1_ + 2×n_2_ + 3×n_3_ + 4×n_4_ + 5×n_5_) / n_total_, where n_total_ is the total number of flies and n_x_ is the number of flies that reached zone X. One experiment consisted of 3 trials performed at 5 min intervals. Two or three independent experiments were performed for each condition, and the mean climbing indexes of independent experiments are shown. All climbing assays were performed 2 hr after lights on (ZT2) to avoid any circadian variation in locomotor activities.

### L-dopa treatment

The feeding of the flies with L-dopa feeding was performed as described previously with minor modifications [61]. Flies were raised on fresh medium made from instant food (formula 4–24) containing the antioxidant ascorbic acid (25 mg/100 ml), the antifungal agent Nipagin and L-dopa (1 mM) (Sigma Aldrich, D9628). Control vials contained only ascorbic acid and Nipagin.

### Immunostaining

For the co-immunostaining of fly brains with anti-GFP and nc82 antibodies, flies were decapitated and the heads were fixed with 4% paraformaldehyde +0.3% Triton X-100 for 1 hour on ice and washed twice with PBST-0.5 (PBS, 0.5% Triton X-100). Subsequently, the head cuticle was partly removed and the heads were washed twice more and blocked in blocking solution (5% normal goat serum, PBS, 0.5% Triton X-100) for 1 hr at room temperature and incubated with the primary antibodies overnight at 4°C. After 2 washes, the heads were incubated with secondary antibodies (Alexa-conjugated) for 2 hours at room temperature. Cuticles and tracheas were removed, and the brains were mounted in Vectashield mounting medium. For staining with anti-TH antibodies with or without other antibodies, 0.3% Triton X-100 was added instead of 0.5% in PBST and in blocking solution. Primary antibodies were incubated over 2 nights at 4°C, and secondary antibodies were incubated at 4°C overnight. The primary antibodies and concentrations used in this study were as follows: rat monoclonal anti-GFP (GF090R) (Nacalai Tesque, Inc.) 1∶500, rabbit polyclonal anti-tyrosine hydroxylase (ab152) (Millipore) 1∶100, mouse monoclonal antibody nc82 (Developmental Studies Hybridoma Bank) 1∶100, mouse monoclonal anti-RFP (AKR-021) (Cell BioLabs, INC.) 1∶500, mouse monoclonal anti-tyrosine hydroxylase (22941) (Immunostar) 1∶50 and polyclonal rabbit anti-GFP (A6455) (Invitrogen) 1∶200.

### Microscopy and image analysis

Leica TCS SP5 confocal microscope was used to image fly brains. Quantification was performed using ImageJ software (NIH). To count the number of DA neurons, anti-TH-positive neurons or anti-TH, anti-GFP double-positive neurons were counted manually through confocal Z-stacks. To image the expression of hlh-13-GFP a Leica DM5500 B microscope was used. TILL Phototonics iMIC digital microscope was used to image DA neurons in worms and DA neurons were counted manually on each Z-stack using ImageJ software.

### qRT-PCR

Total RNA isolation from fly heads, cDNA synthesis and quantitative-PCR (qPCR) analysis were performed as described previously [17]. mRNA levels of *Fer2* were normalized to those of the housekeeping gene *elongation factor 1β* (*Ef1β*). For qPCR analysis of worm mRNAs, worms were collected from the plates with M9 buffer, placed into the Falcon tubes and left to settle for 15 min to remove bacteria in their guts. Worms were then washed twice by spinning at 3000 rpm for 1 min. Total RNAs were isolated using TRIZOL (Life Technologies), and mRNAs were reverse-transcribed and used as templates for qPCR. *hlh-13* mRNA levels were normalized to the *its-1* levels.

### Statistical analyses

Statistical analyses were performed using StatPlus (AnalystSoft) and SPSS software (IBM). For normally distributed data sets, two-tailed Student's t-tests were used to compare the means of two groups. The data that were not normally distributed were analyzed with non-parametric statistics (Mann–Whitney U test). For all experiments, the level of significance was set at p<0.05.

### Sample sizes

The numbers of brain hemispheres examined in Figure 4A-D are as follows. (A) Control (*Fer2^2^/+*): day 0, n = 6; day 1, n = 9; day 21, n = 8. *Fer2^2^*: day0, n = 6; day1, n = 6; day 21, n = 9. (B) *Fer2 > miR Fer2-N*: day 0, n = 6; day 35, n = 9; day 49, n = 17. *Fer2 > miR Fer2-4*: day 0, n = 8; day 35, n = 7; day 49, n = 19. *Fer2 > miR Fer2-5*: day 0, n = 8; day 35, n = 7; day 49, n = 21. (C) *R58E02 > miR Fer2-N*: day 0, n = 10; day 21, n = 8; day 35, n = 12; day 63, n = 14. *R58E02 > miR Fer2-4*: day 0, n = 8; day 21, n = 12; day 35, n = 16; day 63, n = 16. *R58E02 > miR Fer2-5*: day 0, n = 10; day 21, n = 8; day 35, n = 14; day 63, n = 17. (D) Control without UAS-transgene (*Fer2-GAL4*, *tub80*): day 0, n = 15; day 28, n = 7; day 35, n = 18. Control miRNA (*Fer2 > miR Fer2-N*): day 0, n = 15; day 28, n = 13; day 35, n = 24. *Fer2 > miR Fer2-4*, *tub80*: day 0, n = 19; day 28, n = 14; day 35, n = 18. *Fer2 > miR Fer2-5*, *tub80*: day 0, n = 13; day 28, n = 20; day 35, n = 13.

Sample sizes in Figure 5B are as follows. Positive control (*Fer2^2^/+*): day 0, n = 8; day 14, n = 14. Negative control (*Fer2^2^*, *R58E02-gal4*): day 0, n = 15; day 14, n = 16. Rescue (*Fer2^2^*, *R58E02 > Fer2*): day 0, n = 17; day 14, n = 13.

### H_2_O_2_ treatment

5-day-old flies were transferred into the empty vials for 6 hrs, then placed onto the food prepared from instant food (Formula 4–24 Instant Drosophila Medium, Carolina(R)) containing 5% H_2_O_2_ for 24 hrs. Control food contained only dH_2_O. The vials were placed in a humid box and kept at 25°C. Flies were subsequently collected for RNA analysis or placed on the normal food for 24 hrs prior to the dissection and TH staining.

Worms were synchronized by bleaching and stressed as young adults 2 days later (22.5°C). After washing worms off the plates with M9 medium, they were spun down gently at 130 g and washed once with M9 to remove bacteria in the solution. The worms were transferred in 5 ml M9 to an empty petri dish and H_2_O_2_-containing M9 (5 ml) was added to the final concentration of 1 mM. The worms were kept shaking for 30 min at room temperature. Worms were then washed 3 times with M9 to remove H_2_O_2_ and were placed back on the normal NGM plates for recovery.

### ROS detection

*In vivo* detection of ROS production in fly brains was performed using 2′7′-dichlorofluorescein (H2DCF) as detailed in Owusu-Ansah et al. (*Protocol Exchange*, 2008, doi:10.1038/nprot.2008.23). Brains of the *R58E02-GAL4*, *UAS-mCherry* flies in the *Fer2^2^* heterozygous or homozygous background were imaged by confocal microscopy and the fluorescence intensity was measured using the FIJI software [62]. Since there was no regional specificity in the H2DCF signal, the central brain area (entire brain except for the optic lobe) was manually defined and the signal intensity in the defined region across Z-stacks was measured by performing a Z-SUM projection. To quantify the H2DCF signal within PAM neurons, PAM neurons were defined by thresholding the RFP signal and the total H2DCF signal within the defined volume was measured by a Z-SUM projection.

## Supporting Information

Figure S1 *Fer2^1^* mutation does not affect the apparent morphology of major neuron types. *UAS-GFP* was expressed in the control (*Fer2^1^/+*) and *Fer2^1^* flies in cholinergic neurons with *Cha-GAL4*, glutamatergic neurons with *Dvglut-GAL4*, and serotonergic neurons with *TRH-GAL4*. Brains of the 7-day-old flies were stained with anti-GFP and nc82 antibodies. Representative confocal z-projection images are shown. At least 10 brains per genotype were examined and showed no apparent morphological differences between the control and *Fer2^1^* flies. Scale bar, 50 µm.
(TIF)

Figure S2 Loss of PAM and PAL neurons in *Fer2^1^* mutants. (A) Brains of the control (*Fer2^1^/+*) and *Fer2^1^* flies expressing *UAS-GFP* with *HL9-GAL4* were stained for GFP and TH. Representative images of anterior brain regions including the PAM and PAL clusters are shown. Scale bar, 10 µm. (B) Brains of the *Fer2-GAL4*, *UAS-RFP* flies stained for RFP and TH. Since PAM neurons are numerous and densely positioned, only a part of the PAM cluster is shown for clarity. Arrows indicate 5 PAL neurons. Scale bar, 10 µm. (C) Quantification of the TH-immunoreactive PAM and PAL neurons in *Fer2^1^/+* (n = 19), *Fer2^1^* with *GAL4* only (n = 11) and *Fer2^1^* expressing *UAS-TH* with *Fer2-GAL4* (n = 10). *Fer2> TH* did not rescue the number of TH-positive neurons in *Fer2^1^*, indicating the absence of PAM and PAL neurons in the *Fer2^1^* flies (day 0). Mean ± SEM, **p<0.01. ***p<0.001. (D) A schematic of the genetic tracing of a *GAL4*-expressing lineage, showing *Fer2-GAL4* as an example. (E) Representative images of the *Fer2-GAL4* lineage marked by GFP expression in 7-day-old control (*Fer2^1^/+*) and *Fer2^1^* flies. In *Fer2^1^*, PAM neurons were largely undetectable and there were no ectopic GFP-positive neurons. (F) Quantification of PAM and PAL neurons examined by TH/GFP double staining in the *Fer2-GAL4*-expressing lineage. Mean ± SEM. ***p<0.001, *p<0.05, comparing *Fer2^1^/+* and *Fer2^1^* or *Fer2^1^* day 0 vs. day 7.
(TIF)

Figure S3 *Fer2^1^* mutation impairs the development of PAM and PAL neurons. (A) Brains of the *Fer2^1^/+* and *Fer2^1^* pupae 2 and 5 days after puparium formation (APF) stained with anti-TH. Bottom panels are high-magnification images of the PAM neurons in the squares shown in the top panels. White arrows indicate the examples of matured PAM neurons having cytoplasmic TH expression. Yellow arrowheads point to unknown cells weakly expressing TH. Because these cells were found also in other brain areas and only at day 2 APF, they were excluded from the analysis. Scale bars, 50 µm (top) and 5 µm (bottom). (B) Number of matured PAM and PAL neurons in the *Fer2^1^/+* and *Fer2^1^* pupae. Mean ± SEM. ***p<0.001.
(TIF)

Figure S4 Loss of PAM neurons in *Fer2^2^* mutants. (A) Quantification of PAM neurons in the *HL9-GAL4*-expressing lineage in the *Fer2^2^/+* and *Fer2^2^* background. Significantly fewer PAM neurons were detected in *Fer2^2^*. Mean ± SEM. ***p<0.001. *Fer2^2^/+*, n = 12. *Fer2^2^*, n = 12. (B) *Fer2 > Fer2-FLAG* genetic rescue restored the number of PAM neurons in the *Fer2^2^* flies (7-day-old). Mean ± SEM. ***p<0.001. *Fer2^2^/*+, n = 7. *Fer2^2^, Fer2-gal4*, n = 8. *Fer2^2^, Fer2> Fer2*, n = 6.
(TIF)

Figure S5 *Fer2* knockdown leads to a progressive loss of PAM neurons. (A) *Fer2* knockdown efficiency analyzed by qPCR. *Fer2* mRNA levels in the flies expressing *miR Fer2s* and negative control *miR-Fer2-N* using *Fer2-GAL4* were normalized to the level in *w^1118^*. (B) Quantification of PAM neurons in the flies with a constitutive expression of *miR Fer2s* by *HL9-GAL4*. Box boundaries are the 25th and 75th percentiles, the horizontal line across the box is the median, and the whiskers are the lowest and highest values that were not outliers. The open circles represent outliers. *HL9 > miR Fer2-N*: day 0, n = 7; day 21, n = 9; day 63, n = 6. *HL9 > miR Fer2-4*: day 0, n = 6; day 21, n = 9; day 63, n = 6. *HL9 > miR Fer2-5*: day 0, n = 7; day 21, n = 7; day 63, n = 12. *p<0.05, comparing *miR Fer2-N* and *miR Fer2-4 or -5* at the same age. Although PAM neuron counts between *miR-Fer2-N* and *miR-Fer2-5* at day 63 were not significantly different (p = 0.08), *miR-Fer2-5* between day 0 and day 63 were significantly different (p<0.01), suggesting a gradual loss of PAM neurons in *HL9 > miR-Fer2-5*.
(TIF)

Figure S6 Selective vulnerability of PAM neurons in *Fer2* loss-of-function mutants. (A) Mitochondrial morphology in PAM neurons visualized by expressing *mitoGFP* in the *Fer2^2^* and the heterozygous control flies (14-day-old). Scale bar, 5 µm. (B) Mitochondrial morphology in serotonergic neurons in *Fer2^1^* and *Fer2^1^*/+ was monitored by expressing *mitoGFP* with *TRH-GAL4* (7-day-old). Panels 1 and 2 are high magnification images of the areas 1 and 2 in the left panels. Scale bars, 50 µm (left) and 5 µm (panels 1 and 2). (C) Number of DA neurons except for PAM neurons in the *Fer2^2^* flies with or without 24-hr H_2_O_2_ treatment. Mock, n = 32. H_2_O_2_, n = 24. There were no statistically significant differences between mock and H_2_O_2_-treated groups in any cell type.
(TIF)

Figure S7 *hlh-13* is not required for the basal slowing response in worms. (A) The basal slowing response of wild-type and *hlh-13(tm2279)* worms scored by manually counting body bends. (in) and (out) indicate the worms in or out of the food, respectively. No significant differences were observed between the two genotypes at any age examined (Student's t-test). Mean ± SD. (B) The basal slowing response of wild-type and *hlh-13(tm2279)* worms after H_2_O_2_ treatment. Worms were video-recorded and average speed of approximately 30 worms per group was analyzed. Data are shown as a mean from 5-6 independent experiments ± SD. No significant difference between the two genotypes was found by Student's t-test.
(TIF)

Text S1 Materials and methods for basal slowing response in worms.
(DOCX)
