# Supplementary figures and images for: A Conserved Role for p48 Homologs in Protecting Dopaminergic Neurons from Oxidative Stress
Source: PLoS Genet. 2014 Oct 23;10(10):e1004718. doi: 10.1371/journal.pgen.1004718 (PMC4207665; doi:10.1371/journal.pgen.1004718)

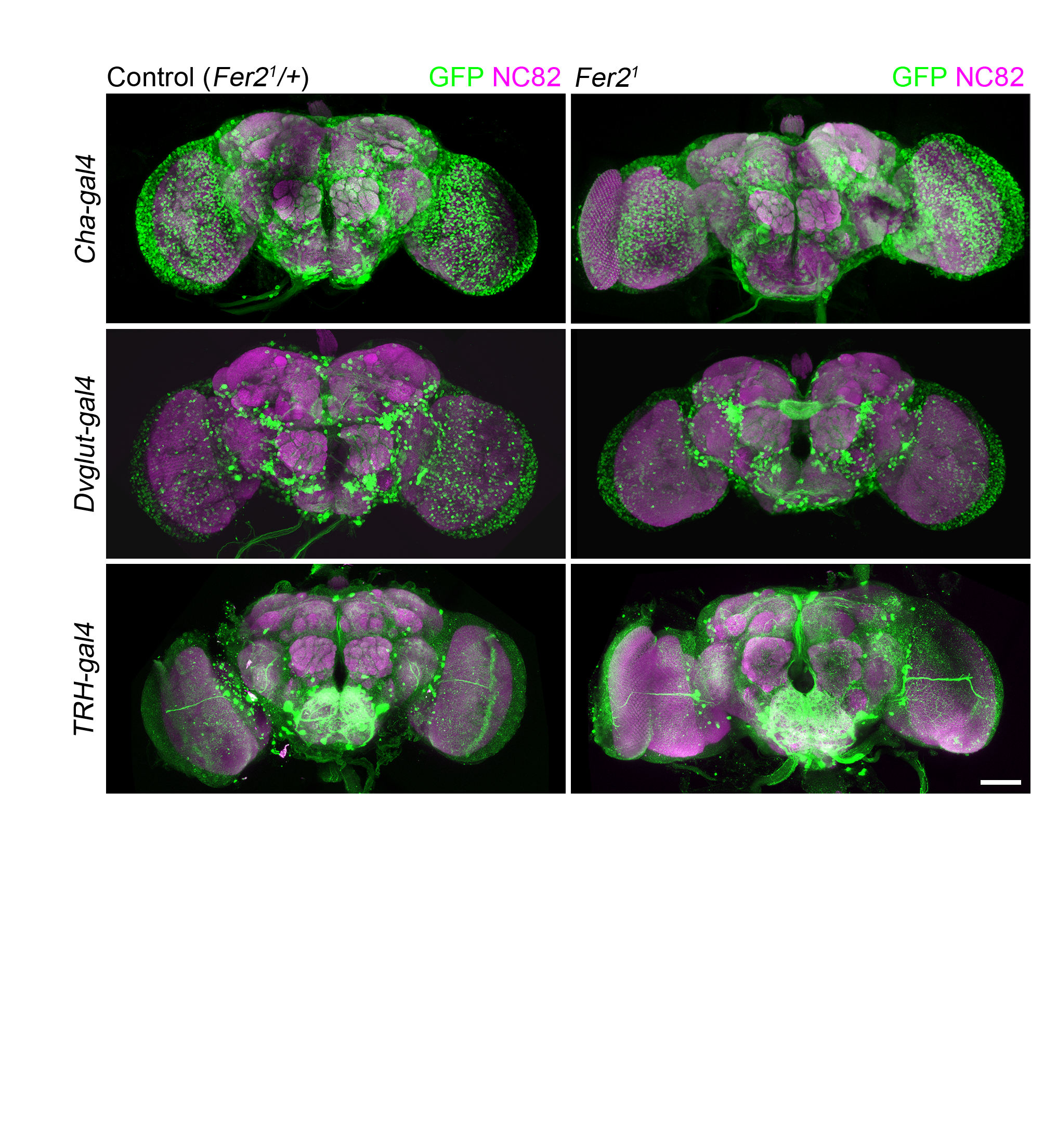

Supplement: Figure S1 — Fer21 mutation does not affect the apparent morphology of major neuron types. UAS-GFP was expressed in the control (Fer21/+) and Fer21 flies in cholinergic neurons with Cha-GAL4, glutamatergic neurons with Dvglut-GAL4, and serotonergic neurons with TRH-GAL4. Brains of the 7-day-old flies were stained with anti-GFP and nc82 antibodies. Representative confocal z-projection images are shown. At least 10 brains per genotype were examined and showed no apparent morphological differences between the control and Fer21 flies. Scale bar, 50 µm. (TIF) [file pgen.1004718.s001.tif]

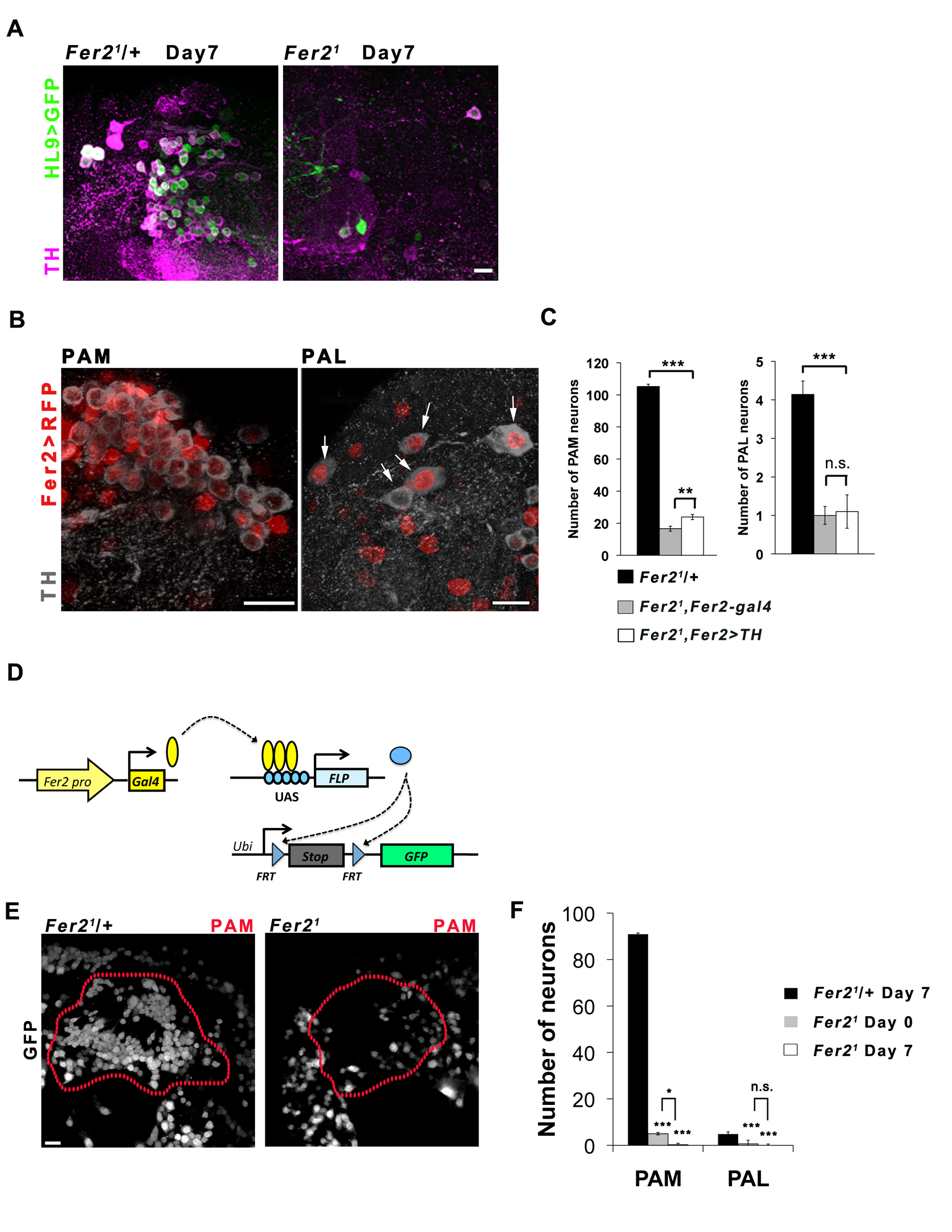

Supplement: Figure S2 — Loss of PAM and PAL neurons in Fer21 mutants. (A) Brains of the control (Fer21/+) and Fer21 flies expressing UAS-GFP with HL9-GAL4 were stained for GFP and TH. Representative images of anterior brain regions including the PAM and PAL clusters are shown. Scale bar, 10 µm. (B) Brains of the Fer2-GAL4, UAS-RFP flies stained for RFP and TH. Since PAM neurons are numerous and densely positioned, only a part of the PAM cluster is shown for clarity. Arrows indicate 5 PAL neurons. Scale bar, 10 µm. (C) Quantification of the TH-immunoreactive PAM and PAL neurons in Fer21/+ (n = 19), Fer21 with GAL4 only (n = 11) and Fer21 expressing UAS-TH with Fer2-GAL4 (n = 10). Fer2> TH did not rescue the number of TH-positive neurons in Fer21, indicating the absence of PAM and PAL neurons in the Fer21 flies (day 0). Mean ± SEM, **p<0.01. ***p<0.001. (D) A schematic of the genetic tracing of a GAL4-expressing lineage, showing Fer2-GAL4 as an example. (E) Representative images of the Fer2-GAL4 lineage marked by GFP expression in 7-day-old control (Fer21/+) and Fer21 flies. In Fer21, PAM neurons were largely undetectable and there were no ectopic GFP-positive neurons. (F) Quantification of PAM and PAL neurons examined by TH/GFP double staining in the Fer2-GAL4-expressing lineage. Mean ± SEM. ***p<0.001, *p<0.05, comparing Fer21/+ and Fer21 or Fer21 day 0 vs. day 7. (TIF) [file pgen.1004718.s002.tif]

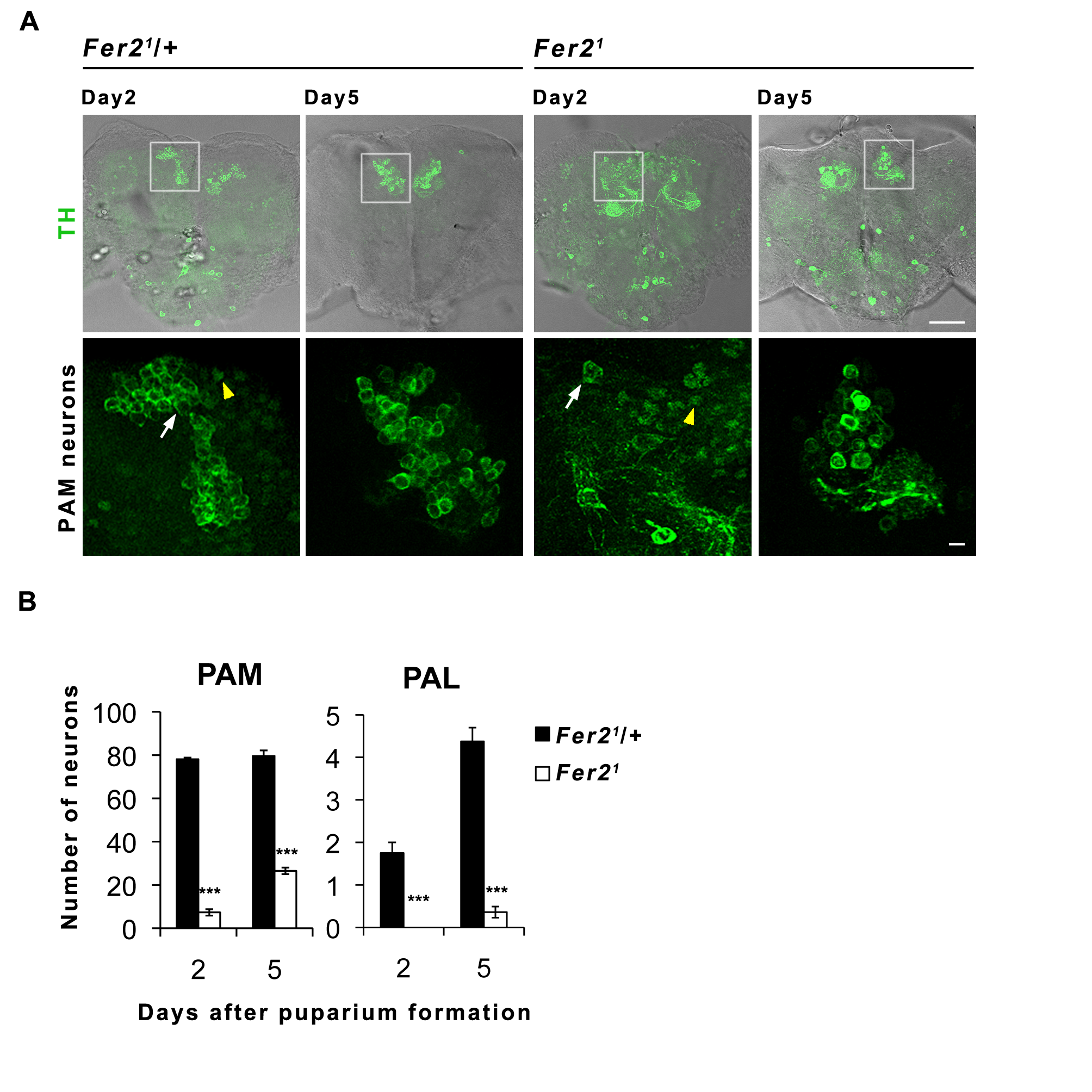

Supplement: Figure S3 — Fer21 mutation impairs the development of PAM and PAL neurons. (A) Brains of the Fer21/+ and Fer21 pupae 2 and 5 days after puparium formation (APF) stained with anti-TH. Bottom panels are high-magnification images of the PAM neurons in the squares shown in the top panels. White arrows indicate the examples of matured PAM neurons having cytoplasmic TH expression. Yellow arrowheads point to unknown cells weakly expressing TH. Because these cells were found also in other brain areas and only at day 2 APF, they were excluded from the analysis. Scale bars, 50 µm (top) and 5 µm (bottom). (B) Number of matured PAM and PAL neurons in the Fer21/+ and Fer21 pupae. Mean ± SEM. ***p<0.001. (TIF) [file pgen.1004718.s003.tif]

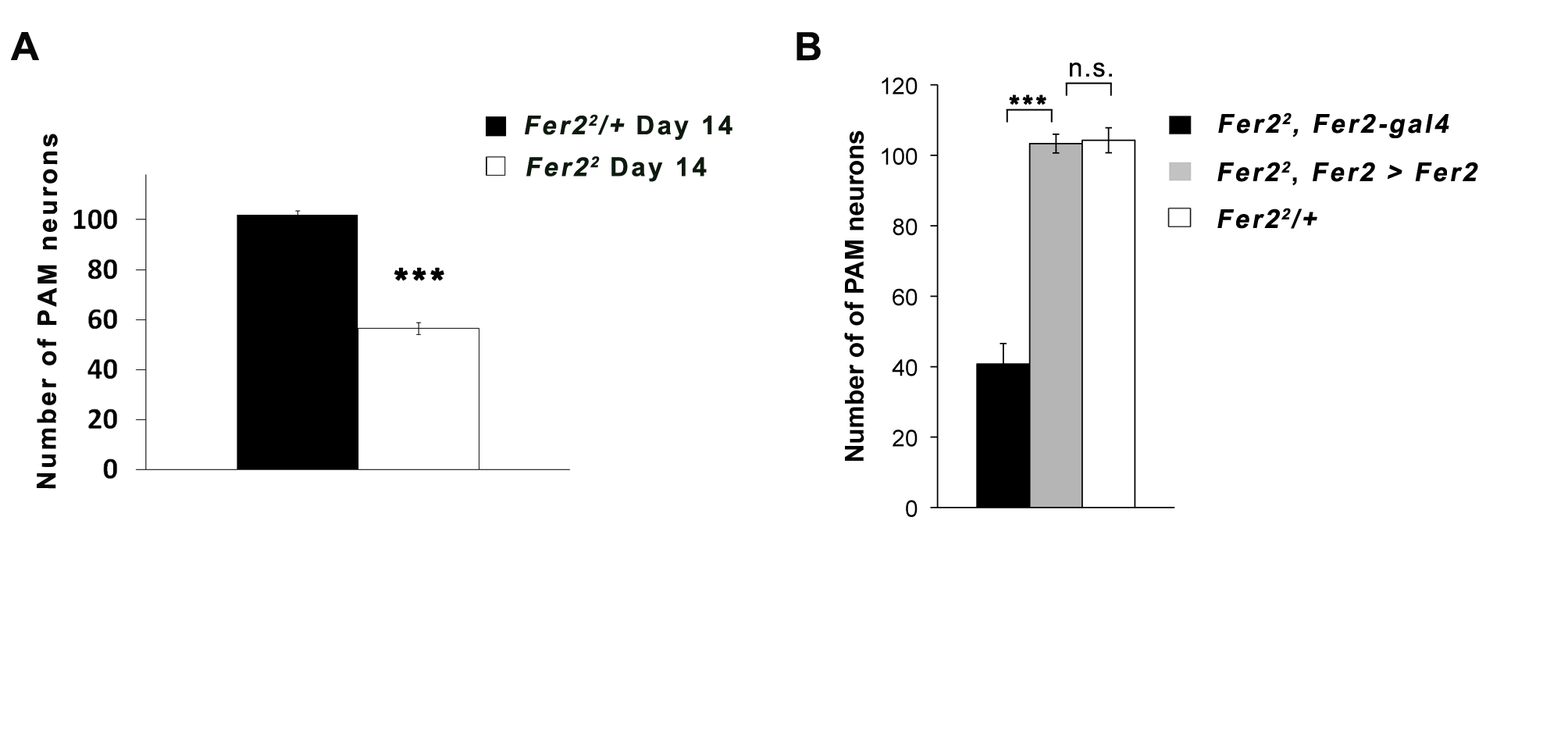

Supplement: Figure S4 — Loss of PAM neurons in Fer22 mutants. (A) Quantification of PAM neurons in the HL9-GAL4-expressing lineage in the Fer22/+ and Fer22 background. Significantly fewer PAM neurons were detected in Fer22. Mean ± SEM. ***p<0.001. Fer22/+, n = 12. Fer22, n = 12. (B) Fer2 > Fer2-FLAG genetic rescue restored the number of PAM neurons in the Fer22 flies (7-day-old). Mean ± SEM. ***p<0.001. Fer22/+, n = 7. Fer22, Fer2-gal4, n = 8. Fer22, Fer2> Fer2, n = 6. (TIF) [file pgen.1004718.s004.tif]

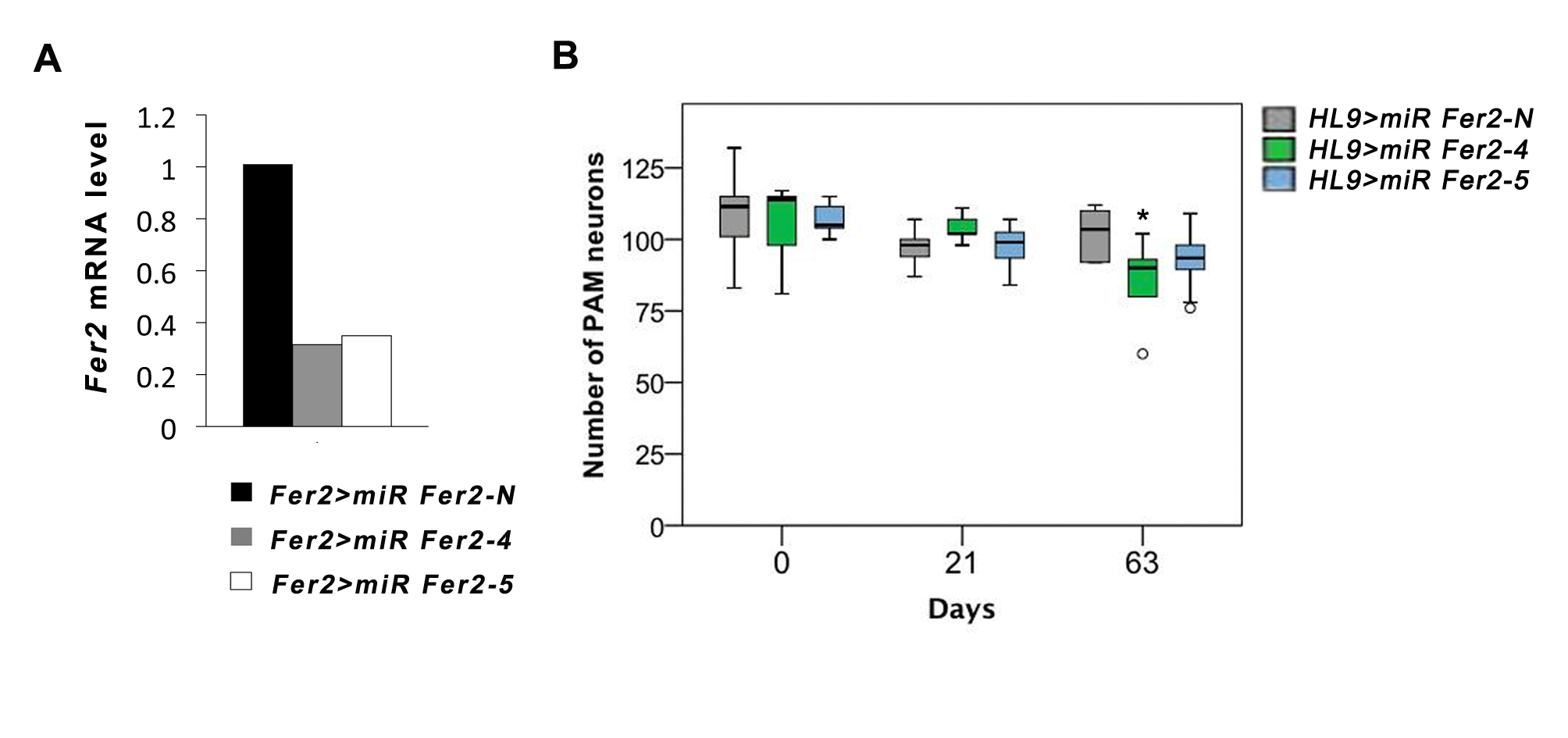

Supplement: Figure S5 — Fer2 knockdown leads to a progressive loss of PAM neurons. (A) Fer2 knockdown efficiency analyzed by qPCR. Fer2 mRNA levels in the flies expressing miR Fer2s and negative control miR-Fer2-N using Fer2-GAL4 were normalized to the level in w1118. (B) Quantification of PAM neurons in the flies with a constitutive expression of miR Fer2s by HL9-GAL4. Box boundaries are the 25th and 75th percentiles, the horizontal line across the box is the median, and the whiskers are the lowest and highest values that were not outliers. The open circles represent outliers. HL9 > miR Fer2-N: day 0, n = 7; day 21, n = 9; day 63, n = 6. HL9 > miR Fer2-4: day 0, n = 6; day 21, n = 9; day 63, n = 6. HL9 > miR Fer2-5: day 0, n = 7; day 21, n = 7; day 63, n = 12. *p<0.05, comparing miR Fer2-N and miR Fer2-4 or -5 at the same age. Although PAM neuron counts between miR-Fer2-N and miR-Fer2-5 at day 63 were not significantly different (p = 0.08), miR-Fer2-5 between day 0 and day 63 were significantly different (p<0.01), suggesting a gradual loss of PAM neurons in HL9 > miR-Fer2-5. (TIF) [file pgen.1004718.s005.tif]

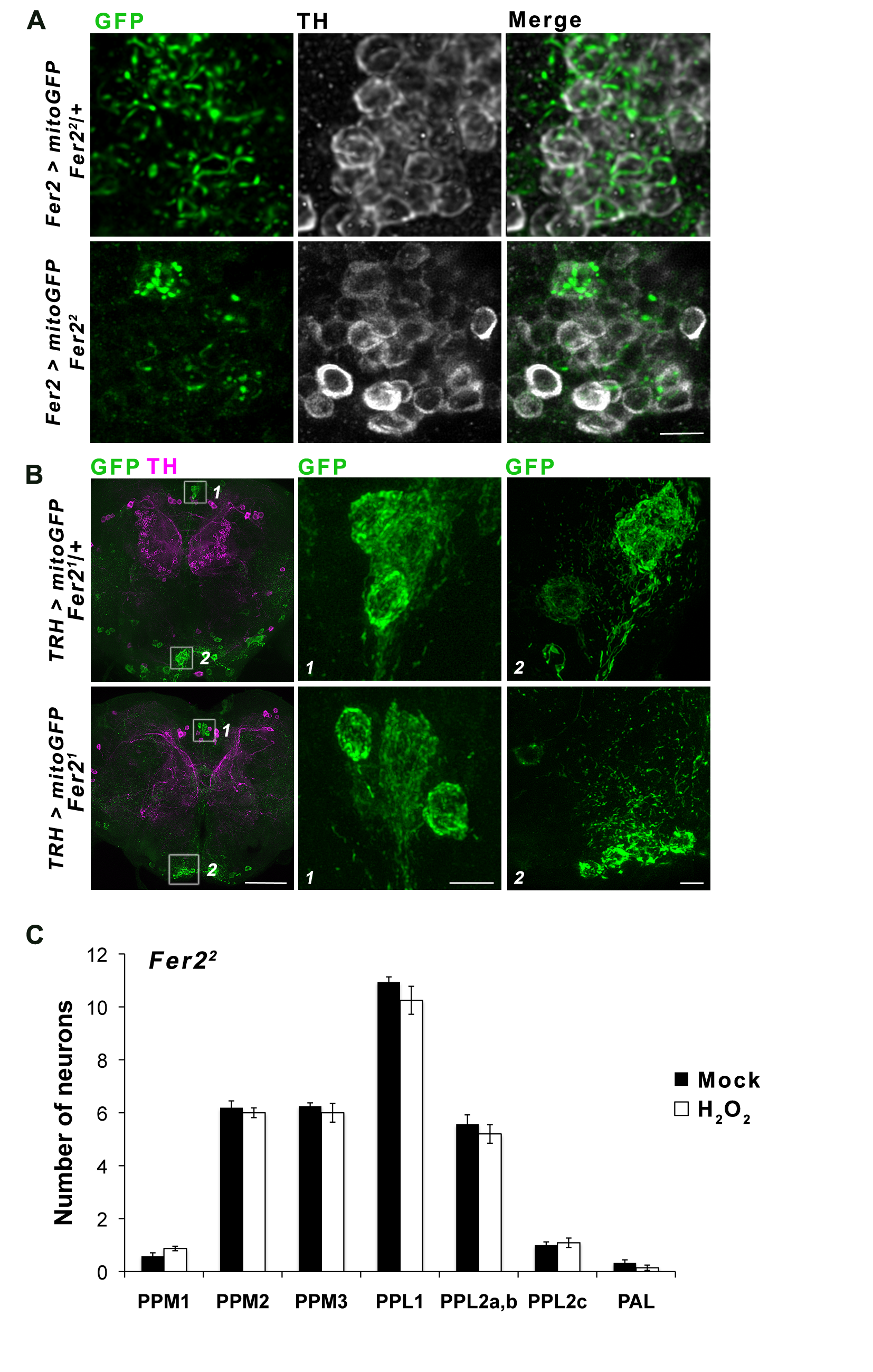

Supplement: Figure S6 — Selective vulnerability of PAM neurons in Fer2 loss-of-function mutants. (A) Mitochondrial morphology in PAM neurons visualized by expressing mitoGFP in the Fer22 and the heterozygous control flies (14-day-old). Scale bar, 5 µm. (B) Mitochondrial morphology in serotonergic neurons in Fer21 and Fer21/+ was monitored by expressing mitoGFP with TRH-GAL4 (7-day-old). Panels 1 and 2 are high magnification images of the areas 1 and 2 in the left panels. Scale bars, 50 µm (left) and 5 µm (panels 1 and 2). (C) Number of DA neurons except for PAM neurons in the Fer22 flies with or without 24-hr H2O2 treatment. Mock, n = 32. H2O2, n = 24. There were no statistically significant differences between mock and H2O2-treated groups in any cell type. (TIF) [file pgen.1004718.s006.tif]

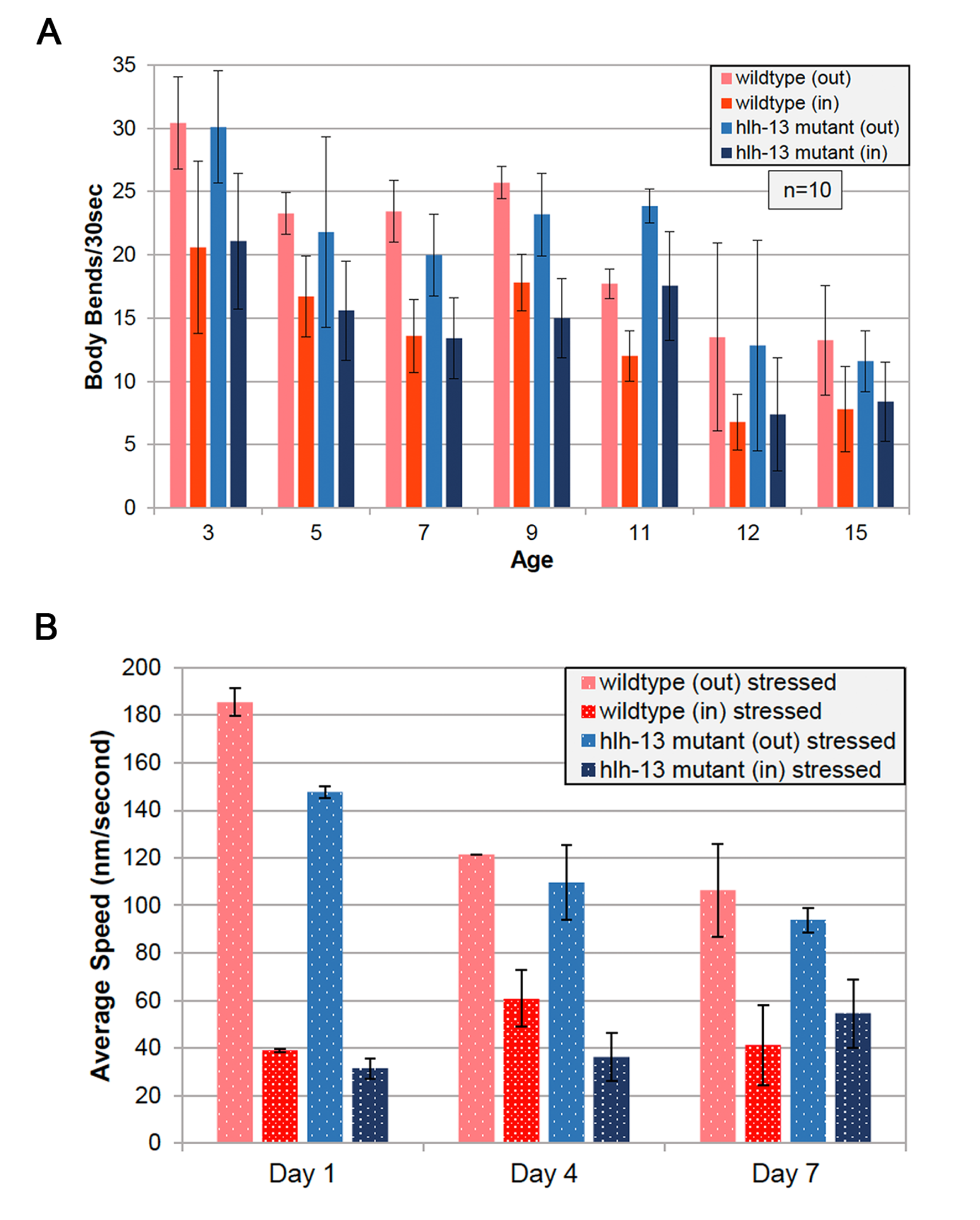

Supplement: Figure S7 — hlh-13 is not required for the basal slowing response in worms. (A) The basal slowing response of wild-type and hlh-13(tm2279) worms scored by manually counting body bends. (in) and (out) indicate the worms in or out of the food, respectively. No significant differences were observed between the two genotypes at any age examined (Student's t-test). Mean ± SD. (B) The basal slowing response of wild-type and hlh-13(tm2279) worms after H2O2 treatment. Worms were video-recorded and average speed of approximately 30 worms per group was analyzed. Data are shown as a mean from 5-6 independent experiments ± SD. No significant difference between the two genotypes was found by Student's t-test. (TIF) [file pgen.1004718.s007.tif]
