# Supplementary material for: A Conserved Role for p48 Homologs in Protecting Dopaminergic Neurons from Oxidative Stress
Source: PLoS Genet. 2014 Oct 23;10(10):e1004718. doi: 10.1371/journal.pgen.1004718 (PMC4207665; doi:10.1371/journal.pgen.1004718)
Supplement: Text S1 — Materials and methods for basal slowing response in worms. (DOCX) [file pgen.1004718.s008.docx]

**Basal slowing response**

Basal slowing response was measured manually or by computer-assisted locomotion analysis. Manual scoring was performed by counting body bends as described previously [1]. For computer-assisted measurements of speed, worms were video-recorded and analyzed with the Multi-Worm Tracker software [2] using the method described in [3] with modifications. Briefly, worms were washed 3 times with distilled water and transferred inside a glycerol coral (2.5 cm x 1.7 cm) placed on a 6 cm NGM 2% agar plate equilibrated at 22 °C with or without bacteria. Worms on plates without bacteria were left for 30 min in order to equilibrate the locomotion rate before recording a video. Worms on plates with bacteria were left for 6 min before recording, according to previously described protocol [1]. The movement of the worms was recorded for 2 min at 7.5 frames per second. Using the Multi-Worm Tracker software, worms were tracked over the full movies and the speed of worms was extracted from the raw data with the use of custom-designed spreadsheets.

**REFERENCES**

1. Sawin ER, Ranganathan R, Horvitz HR (2000) C. elegans locomotory rate is modulated by the environment through a dopaminergic pathway and by experience through a serotonergic pathway. Neuron 26: 619-631.

2. Swierczek NA, Giles AC, Rankin CH, Kerr RA (2011) High-throughput behavioral analysis in C. elegans. Nat Methods 8: 592-598.

3. Schild LC, Glauser DA (2013) Dynamic switching between escape and avoidance regimes reduces Caenorhabditis elegans exposure to noxious heat. Nat Commun 4: 2198.
